# Microstructural changes in human ingestive behavior after Roux-en-Y gastric bypass during liquid meals

**DOI:** 10.1172/jci.insight.136842

**Published:** 2021-08-09

**Authors:** Daniel Gero, Bálint File, Daniela Alceste, Lukas D. Frick, Michele Serra, Aiman E.M. Ismaeil, Robert E. Steinert, Alan C. Spector, Marco Bueter

**Affiliations:** 1 Department of Surgery and Transplantation, University Hospital Zurich, Zurich, Switzerland.; 2 Faculty of Information Technology and Bionics, Pázmány Péter Catholic University, Budapest, Hungary.; 3 Wigner Research Centre for Physics, Budapest, Hungary.; 4 Institute of Cognitive Neuroscience and Psychology, Research Centre for Natural Sciences, Budapest, Hungary.; 5 Department of Psychology and Program in Neuroscience, Florida State University, Tallahassee, Florida, USA.

**Keywords:** Endocrinology, Metabolism, Behavior, Obesity, Surgery

## Abstract

**BACKGROUND:**

Roux-en-Y gastric bypass (RYGB) decreases energy intake and is, therefore, an effective treatment of obesity. The behavioral bases of the decreased calorie intake remain to be elucidated. We applied the methodology of microstructural analysis of meal intake to establish the behavioral features of ingestion in an effort to discern the various controls of feeding as a function of RYGB.

**METHODS:**

The ingestive microstructure of a standardized liquid meal in a cohort of 11 RYGB patients, in 10 patients with obesity, and in 10 healthy-weight adults was prospectively assessed from baseline to 1 year with a custom-designed drinkometer. Statistics were performed on log-transformed ratios of change from baseline so that each participant served as their own control, and proportional increases and decreases were numerically symmetrical. Data-driven (3 seconds) and additional burst pause criteria (1 and 5 seconds) were used.

**RESULTS:**

At baseline, the mean meal size (909.2 versus 557.6 kCal), burst size (28.8 versus 17.6 mL), and meal duration (433 versus 381 seconds) differed between RYGB patients and healthy-weight controls, whereas suck volume (5.2 versus 4.6 mL) and number of bursts (19.7 versus 20.1) were comparable. At 1 year, the ingestive differences between the RYGB and healthy-weight groups disappeared due to significantly decreased burst size (*P* = 0.008) and meal duration (*P* = 0.034) after RYGB. The first-minute intake also decreased after RYGB (*P* = 0.022).

**CONCLUSION:**

RYGB induced dynamic changes in ingestive behavior over the first postoperative year. While the eating pattern of controls remained stable, RYGB patients reduced their meal size by decreasing burst size and meal duration, suggesting that increased postingestive sensibility may mediate postbariatric ingestive behavior.

**TRIAL REGISTRATION:**

NCT03747445; https://clinicaltrials.gov/ct2/show/NCT03747445.

**FUNDING:**

This work was supported by the University of Zurich, the Swiss National Fund (32003B_182309), and the Olga Mayenfisch Foundation. Bálint File was supported by the Hungarian Brain Research Program Grant (grant no. 2017-1.2.1-NKP-2017-00002).

## Graphical abstract

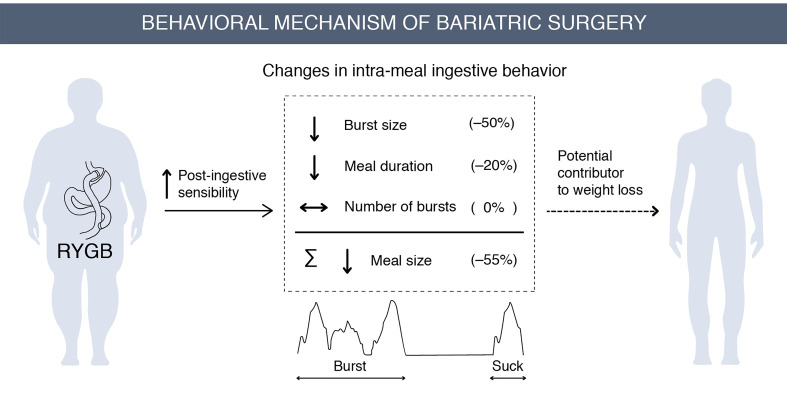

## Introduction

The rising prevalence of obesity worldwide emphasizes the need for improved strategies of prevention and control (1). Bariatric surgery is currently the most effective treatment of severe obesity and associated diseases (2). Roux-en-Y gastric bypass (RYGB) is not only one of the most often performed procedures worldwide (3), but it is also the most intensively investigated procedure to decipher the physiological mechanisms underlying bariatric surgery (4–6).

Potential underlying mechanisms of RYGB include amplified postprandial gut-hormone response (7, 8), changes in vagal nerve signaling mediated by intestinal contents and/or gastrointestinal distention (9), modifications in composition and metabolic activity of gut microbiota (10), and an altered bile acid physiology and signaling through FXR and TGR5 receptors (11).

Additionally, changes in diet selection after bariatric surgery have been suggested as an important candidate mechanism potentiating weight loss (4, 12, 13). According to self-report surveys, patients after RYGB tend to experience earlier satiation, leading to faster meal termination and consequently to smaller meal size (14). However, with few exceptions, most of these findings rely on indirect measures of food intake — e.g., interviews or questionnaires (15, 16) — whereas direct examinations using a cafeteria setting failed to replicate shifts in food preference in humans after RYGB (17, 18). Since self-reported changes may not correlate with measured ingestive behaviors, patients after bariatric surgery may simply consume less of what they were eating before (19). Moreover, the literature is heterogeneous in terms of methodology of sensory techniques and emulation studies, target populations, and lengths of follow-up (16). Therefore, the role of RYGB on food choices in humans remains to be investigated with more direct measures of eating behavior.

Comprehensive understanding of how a given intervention such as RYGB affects food intake requires a detailed analysis of the ingestive behavior itself, not simply the measurement of the outcome of the behavior (18, 20–22). In other words, the information on how the food is consumed is equally or even more important than the information on how much food has been ingested. Consequently, many researchers using rodent models have focused on licking behavior during short-term tests (e.g., 1–60 min), with animals drinking liquid stimuli differing in their chemical properties (e.g., caloric densities, macronutrient composition). This approach allows the microstructural analysis of meal intake, which refers to the temporal distribution and quantitative nature of the most fundamental units of ingestive behavior (e.g., licks, sucks, sips, bites). Microstructural analysis of ingestion depends on the food or fluid source, the animal model, and the time frame of ongoing ingestion (23, 24). Insightful information relevant to distinct motivational modulators of ingestive behavior, such as orosensory input, postoral events, physiological state, and prior experience (i.e., learning/conditioning) can be obtained (25–28).

Recordings of ingestive behavior in humans and rodents after RYGB have produced ambiguous results (14, 22, 29–31), and it remains unclear what drives the decreased overall intake of high-fat and sugary foods after RYGB. There is some evidence from rodent studies suggesting that learning processes may be involved because rats often decrease their fat and sugar preference in a progressive fashion after RYGB (22, 32–35). At issue, however, is whether the decreases in fat and sugar intake/preference, regardless of whether or not they are progressive, are due to changes in the palatability of the stimulus. Although the outcomes of some studies suggest that RYGB blunts the palatability of fat and sugar stimuli (35), others do not (36–38). For example, in one study in which rats displayed progressive decreases in intralipid (a mixture of soya bean oil, egg yolk, glycerin, and water) and sucrose preferences in 48-hour 2-bottle tests and displayed significantly lower preference for these stimuli than sham-operated controls (31), there were no differences between the surgical groups in the breakpoints measured when these stimuli were used as reinforcers in a progressive ratio task. In contrast, in humans, the progressive ratio task showed that RYGB resulted in the selective reduction of the reward value of chocolate candy (sugar + fat) (22), which is consistent with what patients report when they have to rate the desire to consume food items before and after bariatric surgery (39). Adding further complexity to the possible drivers of intake and preference changes is the possibility that there are sensory-discriminative changes in taste sensation. This has been demonstrated by taste-detection thresholds and taste strip tests (21, 40–42), which may reflect changes in food preference after bariatric surgery, although such effects are not always observed (43).

Against this backdrop, it was the aim of this study to assess microstructural changes in ingestive behavior in patients with severe obesity after RYGB in comparison with a healthy-weight and to a nonoperated obese control group. We adopted a translational strategy by applying the methodology and analytical framework of rodents studies to humans. In particular, we deployed a potentially novel custom-designed drinkometer, recently developed and validated by our group (44). In detail, we aimed to map the dynamics of ingestive behavioral adaptations after RYGB by assessing changes in liquid meal intake up to 1 year after surgery to test the hypothesis that the intake of a high caloric liquid meal, as well as the pattern of ingestion within and across meals, changes in a way that contributes to the beneficial outcomes of the surgery.

## Results

### Patients.

**Figure 1:**
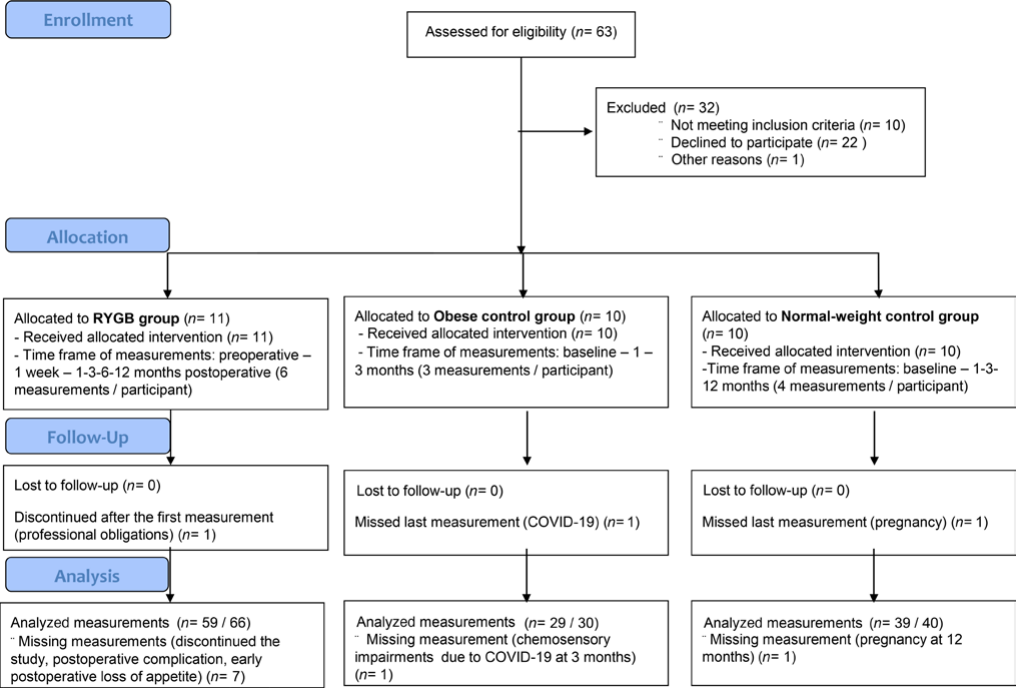
Study flowchart. RYGB, Roux-en-Y gastric bypass.

The 31 participants’ characteristics are presented in Table 1 and the study flowchart in Figure 1. In brief, all participants were nondiabetic female adults aged between 19 and 63 years, not diagnosed with any major eating disorder. The mean BMI at baseline was in the normal range in the healthy-weight control group and > 40 kg/m^2^ in the nonoperated controls and in the patients undergoing RYGB.

### Missing data.

The overall rate of missing data was low (9.3%); 127 measurements were recorded out of the planned 136. In the RYGB group, 1 participant quit the study after the baseline measurement due to professional obligations. Two participants missed the 1-week measurement: 1 person due to a postoperative complication (anastomotic leak) and another person to unwillingness to consume the stimulus. In the healthy-weight control group, 1 participant was excluded from the 1-year measurement due to pregnancy. In the obese control group, 1 participant was excluded from the 3-month measurement due to chemosensory impairments and loss of appetite caused by an acute COVID-19 disease.

### Postoperative weight loss.

**Figure 2:**
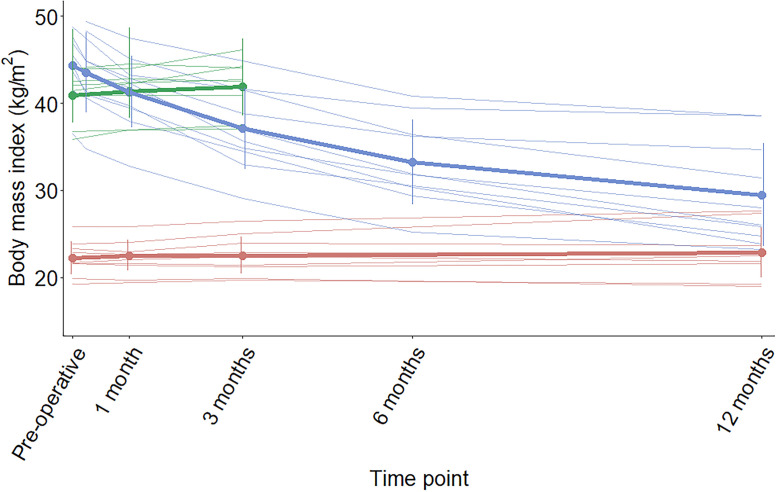
Change in body mass index. Change in body mass index over time expressed as group mean (bold lines) and individual curves (pale lines). Blue, Roux-en-Y gastric bypass group; red, normal-weight control group; green, obese control group. Post hoc test: RYGB baseline versus RYGB 1-year difference: –16.35 (95% CI, –23.15 to –9.5), *P*_adj_ < 0.0001.

The participants’ BMI over time is shown in Figure 2. A 2-way ANOVA showed significant effects of RYGB versus healthy-weight controls (*F*_(1,__87)_ = 358, *P* < 0.001) and time (*F*_(5,__87)_ = 14.2, *P* ≤ 0.001), with significant interaction between these 2 factors (*F*_(3,__87)_ = 16.6, *P* < 0.001). The body weight of the obese controls remained stable over 3 months (*F*_(1,__17)_ = 0.05, *P* = 0.82).

### Identification of an optimal burst pause criterion.

**Figure 3:**
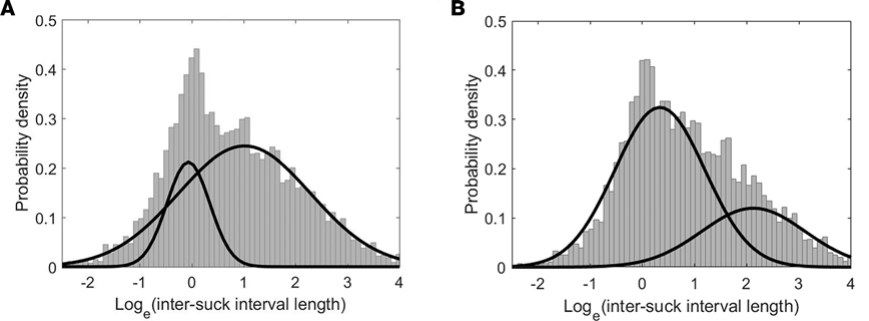
Probability density function (PDF) of log_e_ transformed ISI to identify the optimal burst pause criterion. (**A**) All recorded drinkometer sessions in RYGB patients, normal-weight controls and obese controls. (**B**) RYGB group and normal-weight controls only. Gaussian mixture models aim to distinguish 2 normally distributed populations: the shorter ones represent the ISI, whereas the longer ones represent the IBI. The optimal burst pause criterion between ISIs and IBIs is where the 2 Gaussian curves meet, which was observed at the following: (**A**) 0.122 log_e_ units, representing 1.13 seconds; (**B**) 1.631 log_e_ units, representing 5.1 seconds.

Definitions of bursts, meals, and sucks in this study are based on both our validation study (44), as well as on empirical outcomes with the application of this analytical strategy in rats after RYGB (32). The burst pause criterion (PC) was identified by using probability density function (PDF) with a 2-component Gaussian mixture model fitted to the frequency histograms as previously described (44). Results of the PDF are shown in Figure 3. The mathematically optimal PC in the pooled analysis of the RYGB and healthy-weight control groups was 5.1 seconds; however, the addition of the obese control group led to a PC of 1.13 seconds, despite very similar histogram of intersuck intervals (ISI). To overcome these differences, the median value of 3 seconds was used to create the visual outputs presented in the manuscript. Since the frequency histograms failed to reveal 2 distinct populations of ISI and IBI, and the goodness of fit of the model was not perfect (based on the mathematical method for evaluating how well a model fits the data it was generated from, expressed by the Aikake Information Criterion, which was 13251.8) (45), we additionally analyzed the data at PC of 1 and 5 seconds, to see whether this key analytical feature would fundamentally change the effect of RYGB on the PC-dependent microstructural outcomes. Differences in the PC between bursts have been shown earlier to lead to different microstructural outcomes (23).

### Effects of RYGB on ingestive parameters of the entire meal.

**Figure 4:**
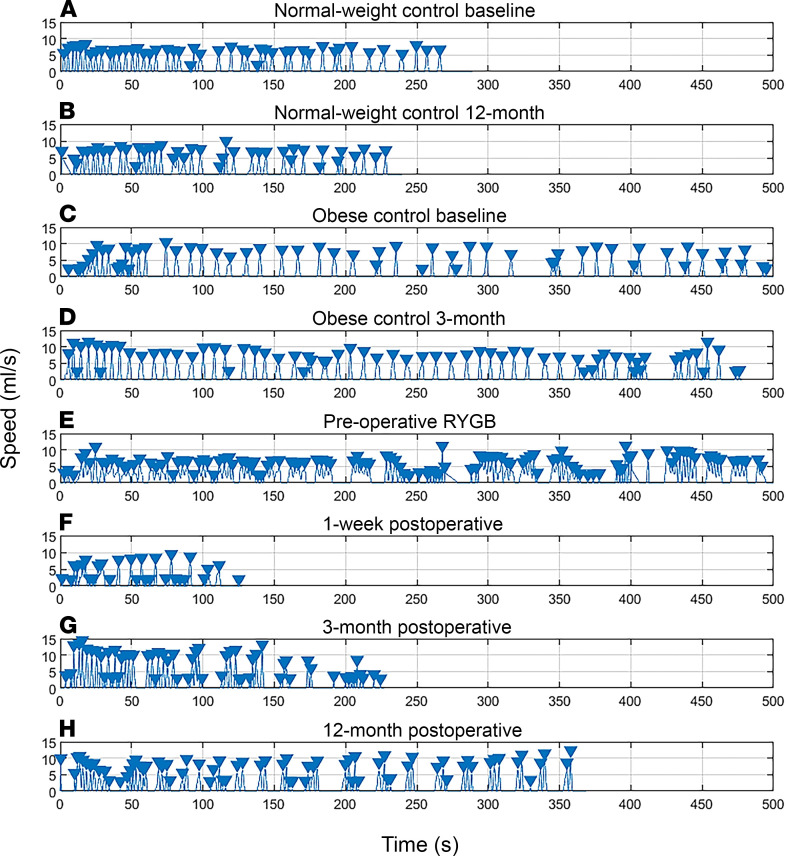
Graphical illustration of different drinking sessions of a single representative participant from each group at different time points. (**A**) Normal-weight control baseline. (**B**) Normal-weight control 12 months. (**C**) Obese control baseline. (**D**) Obese control 3 months. (**E**) Preoperative RYGB. (**F**) One week postoperative. (**G**) Three months postoperative. (**H**) Twelve months postoperative. Triangles show the peak of each suck.

**Figure 5:**
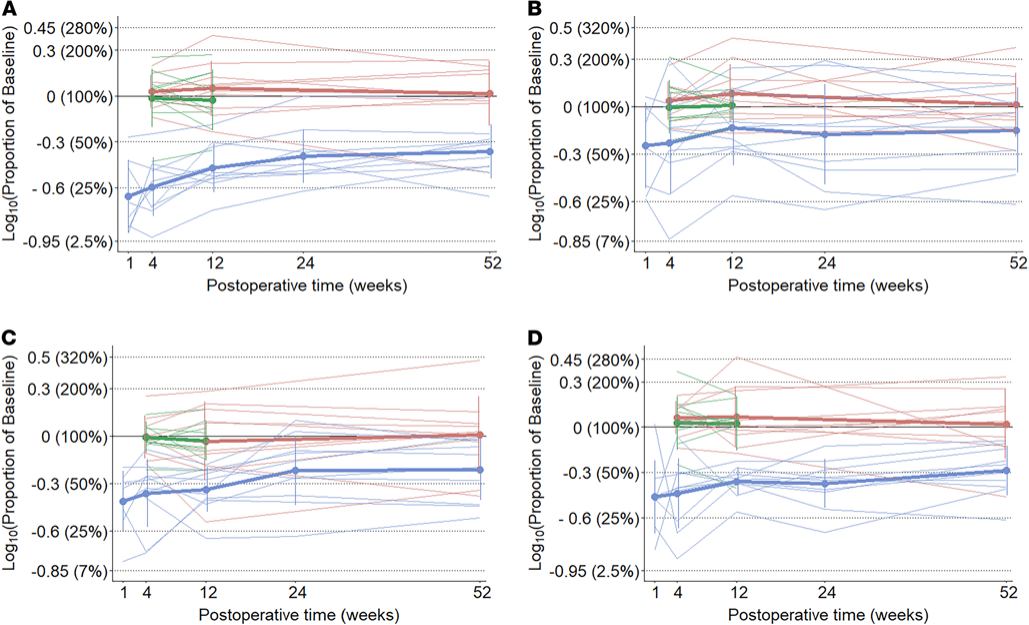
Changes in overall ingestive parameters over time expressed as proportion of baseline. Group means, bold lines; individual curves, pale lines. (**A**) Calorie intake (1 mL of the stimulus contained 2 kCal), post hoc test: RYGB 1 year versus normal-weight control 1 year difference: –0.37 (95% CI, –0.63 to –0.14), *P*_adj_ = 0.0003. (**B**) Meal duration, post hoc test: RYGB 1-month versus normal-weight control 3-months difference: 0.33 (95% CI, 0.01–0.65), *P*_adj_ = 0.036. (**C**) Average drinking speed, post hoc test: RYGB 3-month versus normal-weight control 1-year difference: 0.34 (95% CI, 0.07–0.62), *P*_adj_ = 0.007. (**D**) Total number of sucks, post hoc test: RYGB 1-year versus normal-weight control 1-year difference: –0.3 (95% CI, –0.56–0.06), *P*_adj_ = 0.008. Blue, Roux-en-Y gastric bypass group; red, normal-weight control group; green, obese control group.

**Figure 6:**
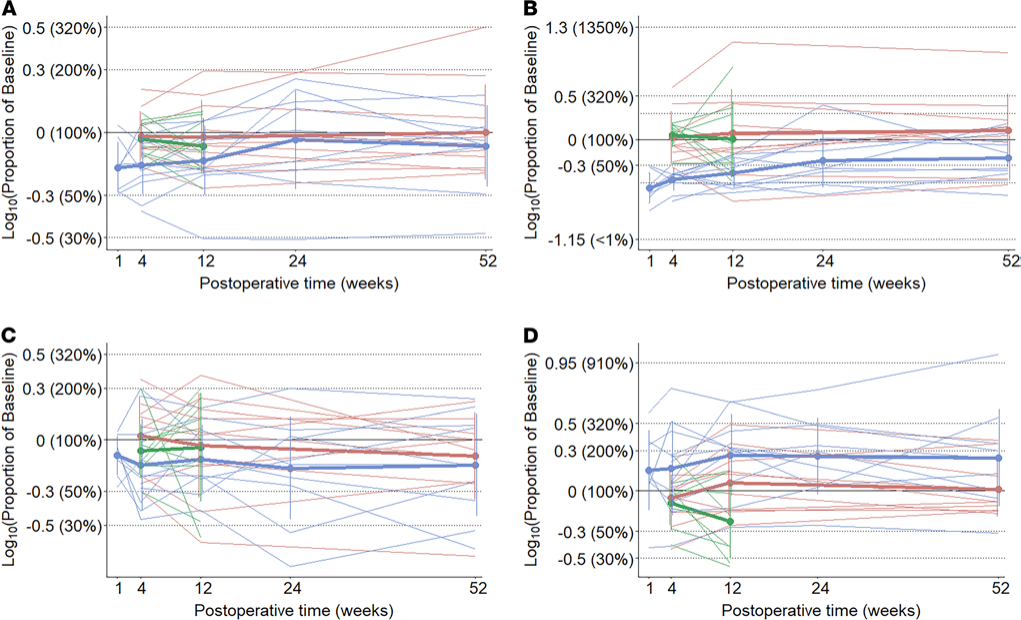
Changes in microstructural parameters over time expressed as proportion of baseline. Group means, bold lines; individual curves, pale lines; burst pause criterion, 3 seconds. (**A**) Suck volume, post hoc test: nonsignificant. (**B**) Mean burst size, post hoc test: RYGB 3-month versus normal-weight control 1-year difference: 0.51 (95% CI, 0.05–0.96), *P*_adj_ = 0.02. (**C**) Total number of bursts, post hoc test: nonsignificant. (**D**) IBI, post hoc test: nonsignificant. Blue, Roux-en-Y gastric bypass group; red, normal-weight control group; green, obese control group.

First, we approached data analysis by visual assessment of each individual drinking session to ensure that observed differences in drinking microstructure did not result from averaging different participants. The microstructure of drinking sessions from 1 representative participant from each group at different time points is shown in Figure 4. Second, we analyzed data after pooling measurements from all drinking sessions. Data related to the overall ingestive parameters are shown in Figure 5 and in Supplemental Figure 1 (supplemental material available online with this article; https://doi.org/10.1172/jci.insight.136842DS1), whereas microstructural parameters are presented in Figure 6 and in Supplemental Figure 2. Characteristics of the entire meal — as well as the number, size, duration, and rate of sucks — are PC independent, whereas the number, size, duration, and rate of sucking bursts and the lengths of within-meal pauses (ISI and IBI) are PC dependent. With regard to the PC-independent parameters, a 2-way mixed ANOVA revealed that RYGB significantly decreased the size, duration, and speed of the entire meal, while the size and speed of sucks remained unaffected (Table 2). With regard the PC-dependent parameters, RYGB had a distinct and significant effect on decreasing the average size and duration of bursts, while the total number of bursts, the average burst rate, the ISI, and the IBI remained stable over time and between groups (Table 3). A group × time interaction was observed only for meal size. Here, the effect of time became nonsignificant for both groups after post hoc adjustment (RYGB, F_(variation between sample means/variation within the samples)_ = 4.19, *P* = 0.052; control, F = 0.04, *P* = 1), while the effect of group remained highly significant at all time points. The 1-way ANOVA performed on the change of ingestive parameters in the obese control group found no significant difference between time points.

### Effect of RYGB on microstructural parameters of the beginning of the meal.

**Figure 7:**
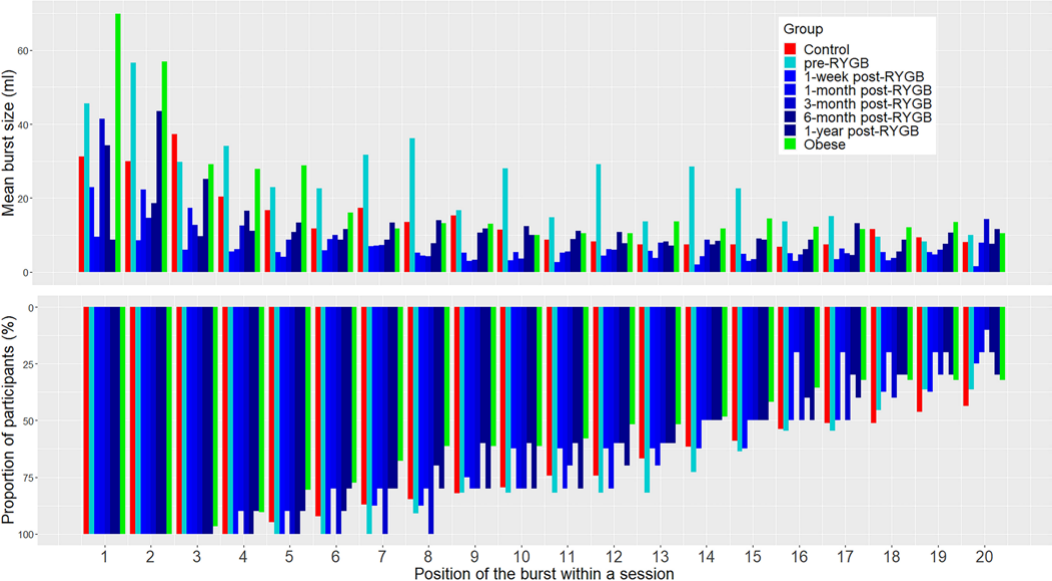
Mean burst size within the first 20 consecutive bursts within a meal according to subgroups. Normal-weight control group (*n* = 39) (red); obese control group (*n* = 29) (green); RYGB group pre-RYGB (*n* = 11); and 1 week (*n* = 8)/1 month (*n* = 10)/3 months (*n* = 10)/6 months (*n* = 10)/1 year (*n* **=** 10) after RYGB (shades of blue) at PC of 3 seconds. The proportion of participants per subgroups and per consecutive burst number is shown in the lower part of the plot.

**Figure 8:**
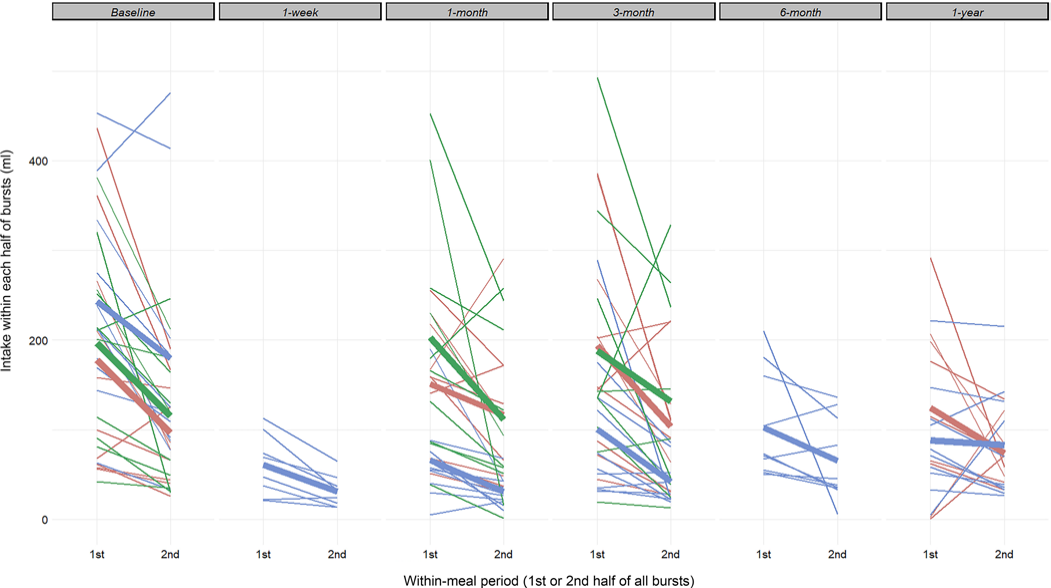
Changes in meal intake between the first and second half of bursts within each ingestive session. Blue, Roux-en-Y gastric bypass group; red, normal-weight control group; green, obese control group.

**Figure 9:**
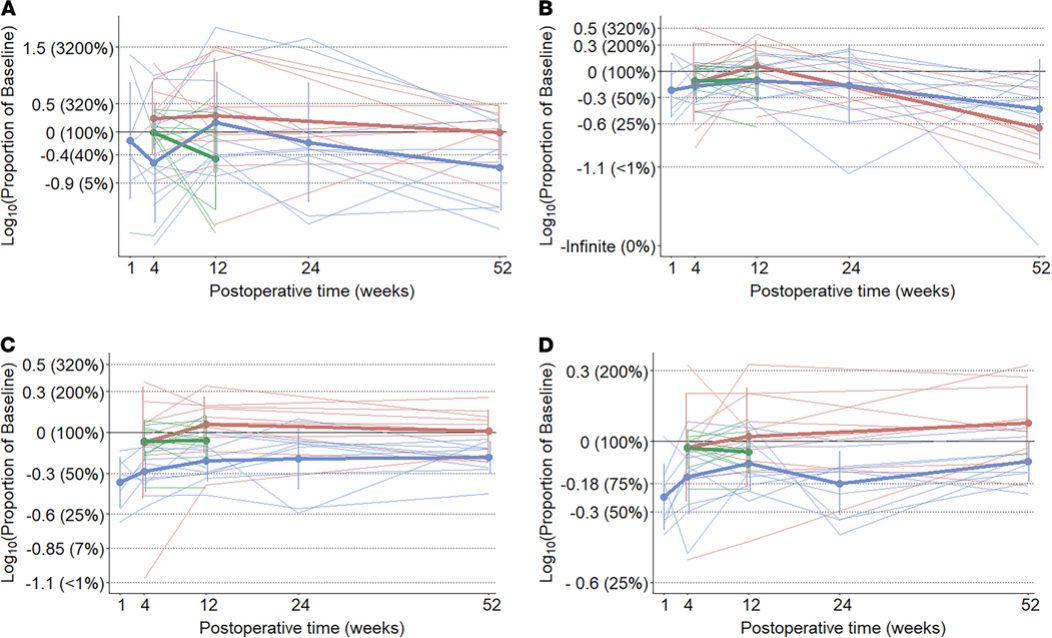
Changes in microstructural parameters at the beginning of the meal expressed as proportion of baseline. Group means, bold lines; individual curves, pale lines. (**A**) Size of the first burst (PC = 3 seconds), post hoc test: RYGB 1-year versus normal-weight control 3-month difference: –1.14 (95% CI, -2.24 to –0.04), *P*_adj_ = 0.04. (**B**) Intake in the first 15 seconds. (**C**) Intake in the first 60 seconds, post hoc test: RYGB 1-month versus normal-weight control 3-month difference: 0.34 (95% CI, 0.04–0.64) *P*_adj_ = 0.02. (**D**) Number of sucks within the first minute, post hoc test: RYGB 1-month versus normal-weight control 1-year difference: 0.24 (95% CI, 0.002–0.466), *P*_adj_ = 0.046. Blue, Roux-en-Y gastric bypass group; red, normal-weight control group; green, obese control group.

First, we visually assessed the mean volume of consecutive bursts across all sessions and found that, in all groups and at all time points, the first 2 or 3 bursts contained the largest volumes (Figure 7). Out of the 127 recorded sessions, with PC set at 3 seconds, only 1 session was organized in 2 bursts, the median was 15 bursts, and the maximal burst number within 1 session was 74 (*n* = 1). The overall intake decreased in both groups at all time points between the first and the second half of bursts (Figure 8). Second, to distill the effect of RYGB on the consumption at the beginning of the meal, we analyzed the intake during the first minute of the meal and within the first burst (Figure 9 and Supplemental Figure 3).

Results of the 2-way mixed ANOVA revealed a significant main effect for RYGB in decreasing intake during the first 60 seconds of the meal, while the intake within the first 15 seconds was more sensitive to the time of the measurement than to the treatment group (PC-independent parameters; Table 2). The size of the first burst changed significantly over time independently of the groups, showing the lack of stability of this parameter (Table 3). There were no significant treatment group × time interactions.

### Self-reported appetite perception.

**Figure 10:**
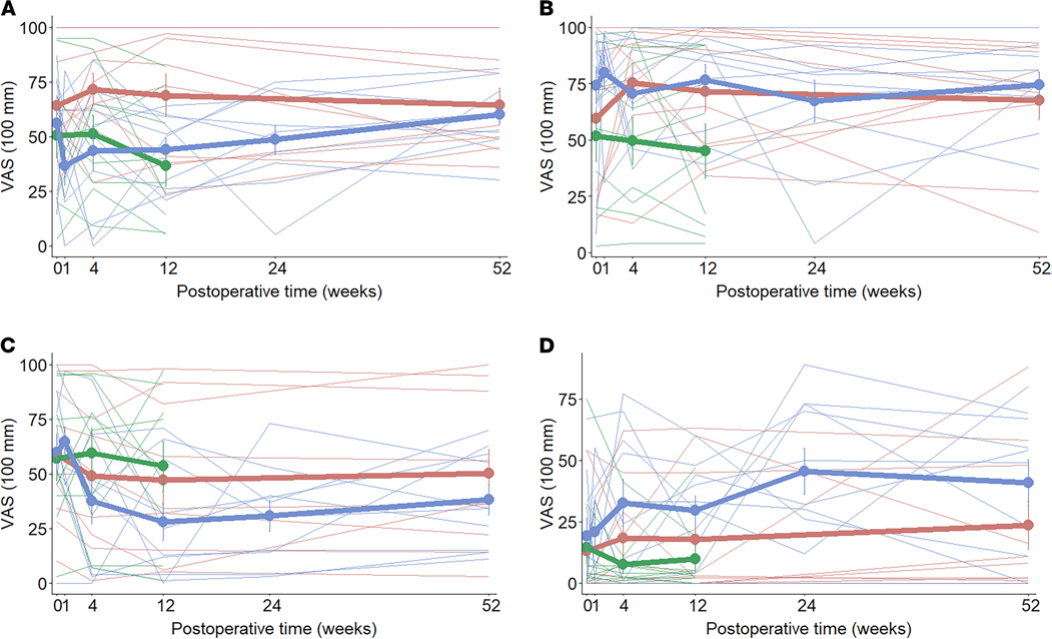
Self-reported periingestive feelings, expressed as absolute values. Group means, bold lines; individual curves, pale lines. (**A**) Premeal hunger. (**B**) Premeal thirst. (**C**) Liking of the stimulus. (**D**) Nausea 30 minutes after the meal session. Blue, Roux-en-Y gastric bypass group; red, normal-weight control group; green, obese control group; VAS, visual analogue scale (100 mm).

Visual interpretation of visual analogue scale (VAS) scores did not show differences in premeal hunger and premeal thirst between the groups at baseline or at 1 year (Figure 10). Both the RYGB and healthy-weight control groups showed a trend of decreased liking of the stimulus and of increased postprandial nausea over time, with a more pronounced change from baseline in the RYGB group. At the completion of the study, if participants could go back in time to the beginning of the study, 10% of healthy-weight and of obese controls and 70% of RYGB patients would have chosen a different flavor for the liquid meal (χ^2^ statistic = 114.29, degrees of freedom [df] = 2, *P* < 0.001).

## Discussion

In this study, we applied a microstructural analysis of a liquid meal intake in patients before and after RYGB, aiming to reveal the behavioral mechanisms that underlie qualitative and quantitative changes in postoperative food intake. We found that preoperative differences in ingestive parameters (i.e., meal size, burst size, or average speed of intake) between patients with obesity and healthy-weight controls vanished by the end of the first year after RYGB. More specifically, patients after RYGB decreased their meal size by 55% within 1 year after the surgery when compared with baseline, which was mainly achieved by decreasing average burst size by 25%–50% (depending on the PC) and overall meal duration by 20%. Thus, the neural circuits that maintain ingestion during a burst are vulnerable to the effects of RYGB, while those responsible for initiating ingestive bursts are relatively impervious to it. Overall, we found that the change in ingestive behavior after RYGB seems to be a dynamic process starting as early as the first week after surgery, rather than being a stable postoperative feature. In fact, the magnitude of changes constantly decreased for most ingestive and microstructural parameters during the first year after RYGB. This observation reflects a gradual postbariatric behavioral adaptation to the physiological changes triggered by the rearranged gastrointestinal anatomy. In patients with obesity in the absence of surgery, we failed to observe any change in ingestive behavior over time, further supporting the causal role of surgery in the induction of the observed behavioral adaptations in the RYGB group.

Preoperatively, the meal size of the RYGB patients was 63% greater compared with the control group. During the follow-up, the meal size of both control groups remained stable, while in the RYGB group, we observed a decrease. The smallest meal size was measured 1 week after RYGB, followed by a constant increase thereafter resulting in a similar meal size as in healthy-weight controls at the end of the first postoperative year. This is in line with previous reports also demonstrating a rather drastic meal size reduction early after RYGB, followed by a stepwise increase within the first postoperative year (46).

Average burst size showed a similar trajectory of post-RYGB adaptation as meal size. In rats, average burst size increases monotonically as a function of sucrose concentration (23, 26), and it decreases in thirsty rats when quinine replaces water (27). It is therefore thought to be heavily influenced by the orosensory properties of the stimulus. Furthermore, burst size decreases as the meal progresses, reflecting the influence of the accumulation of postingestive load (23). In our study, the size of consecutive bursts within meals tended to decline in all groups at all time points, suggesting that, as the meal approaches termination, burst size progressively decreases in humans also, especially in the second half of bursts, which likely reflects the onset of satiation. Interestingly, patients before RYGB presented an additional surge in burst size in the middle of the meal, which was not present postoperatively or in controls. The total number of bursts averaged around 15 and was not significantly affected by RYGB, suggesting that RYGB lowers overall intake by affecting processes that control burst termination, but not those that control burst initiation. It is interesting that the processes that initiate bursts do not compensate for the decreased ingestion; instead, they stay stable over time and comparable between the groups.

**Figure 11:**
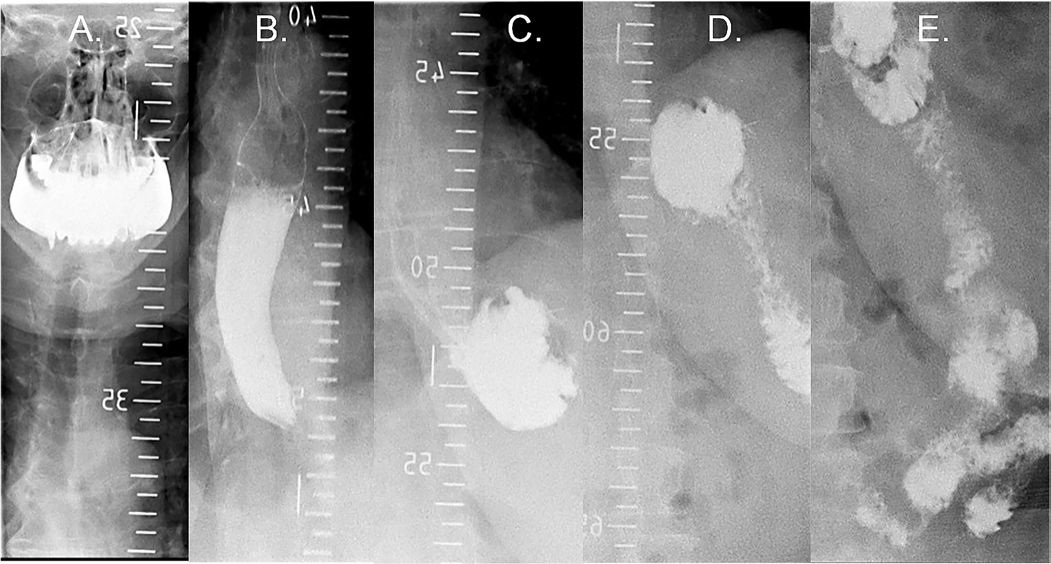
Oral contrast material swallow series in a patient after Roux-en-Y gastric bypass. (**A**) Baseline: oral cavity is filled. (**B**) At 4 seconds: contrast present in distal esophagus. (**C**) At 10 seconds: contrast fills the gastric pouch. (**D**) At 12 seconds: contrast reaches the alimentary limb. (**E**) At 45 seconds: contrast present in the common channel.

In the first minutes of a drinking episode, the behavior is more under control of the orosensory properties of the stimulus because it precedes any significant fluid accumulation in the stomach or small bowel (25, 26). In our study, the first-minute intake and the size of the first burst decreased early after surgery and remained more or less stable throughout the entire observation period. Of note, in the RYGB group, both the average size of the first burst and the ingested volume during the first minute were larger than the reservoir capacity of the gastric pouch after RYGB (estimated around 25–30 mL). This suggests that at least a portion of the ingested fluid had already entered into the small bowel within the first minute of the meal in patients after RYGB, while in the control group, the ingested volumes during the respective meal stage were likely to have been harbored in the oral cavity, esophagus, or stomach. Human RYGB consists of the creation of a small (~25 mL) gastric pouch that is directly connected to the jejunum. Nutrients bypass the remnant stomach, duodenum, and proximal jejunum and are delivered directly into the distal jejunum. Contrast material studies of the upper gastrointestinal tract in patients with RYGB show that liquids reach the alimentary limb within 15 seconds after the beginning of the act of swallowing (Figure 11 and Supplemental Video 1). Biliopancreatic fluid is delivered to the common intestinal channel via a jejuno-jejunal anastomosis. Due to the decreased storage capacity of the gastric pouch and exclusion of the pylorus muscle from the gastrointestinal circuit, the half-time of gastric pouch emptying is reduced to minutes, leading to an increased intestinal caloric delivery rate, which can reach 100 kCal/min for liquid meals (47). Accelerated intestinal food delivery is thought to impact subsequent gastrointestinal motor, neural, and hormone functions profoundly, and it may explain at least part of the observed efficacy of RYGB surgery on eating behavior.

Therefore, we assume that, after RYGB, the size of the first burst not only depends on palatability, but it may also be influenced by very early postingestive signals. Thus, the physiologic readout of the first burst size/first-minute intake may be different in patients after RYGB simply because the food enters the small bowel much faster than in subjects without an altered gastrointestinal anatomy. This, of course, may also be true in rodents that underwent RYGB surgery and suggests that the meaning of established microstructural parameters such as the first minute suck rate or the size of the first burst may have to be reconsidered in the RYGB setting. Previous studies of our own group and others showed that a rapid increase in small intestinal nutrient content after RYGB contributes to the earlier rise of glycemia (7) and to the increased meal-related secretion of CCK, GLP-1, PYY_3–36_, and insulin, which may trigger an earlier perception of satiation (8).

Our findings, therefore, suggest that the reduced meal size following RYGB stems, in part, from signals related to the postingestive load. This is in line with previous findings from our own group in rodents after RYGB, also indicating that the reduced preference for food high in fat and/or sugar after RYGB may be rather related to postingestive signals and subsequent learning, but not a change in palatability (31–33). That said, a potential role for decreases in palatability of the high-fat/high-sugar stimulus cannot be entirely ruled out (23, 26, 27, 48).

The present study also supports the existence of learning processes in postbariatric ingestive behavior. Patients progressively adapted their meal size, which may reflect the countless occasions of self-experimenting the limits of food tolerance outside laboratory conditions. Humans seem to tolerate or even ignore negative postingestive signaling, as both the measured meal intake and the self-reported postprandial nausea increased from one postoperative time point to another. This is in accordance with another study (18) using an ad libitum food intake test, which found no association between patients’ experience of negative responses after eating and food preferences 6 months after RYGB. Furthermore, no change was found in the relative intake of high-fat, low-fat, sweet, and savory foods. However, ingestive microstructure was not measured, and given that only 1 single postoperative time point was reported, the investigators may have missed the dynamic adaptation process described above.

Selection of the optimal PC that defines bursts from an otherwise continuous surge of licking or sucking, is still a matter of debate (23, 49). In this study, we used parallel approaches to bridge this theoretical issue and applied a PDF and a Gaussian mixture model fitted to the frequency histograms of the log_e_ transformed ISI to identify the mathematically optimal PC. In addition, we complemented our analysis by selecting 2 additional PC (at ± 2 seconds from the median of the 2 optimal PCs). This strategy solidified our main observation, that RYGB is associated with a significant decrease in average burst size independent of the chosen PC. Additionally, one may perform analyses using an extreme PC that equals to the entire meal duration to see how the nature of the functions changes as the PC approached the definition of a meal (23). This analysis is the same as the PC-independent analysis of meal size, which was again decreased at all time points following RYGB. On the other end of the spectrum, the duration and volume of ingestive sucks were unaffected by RYGB and remained stable over time in all groups.

Due to their simplicity, self-reported data collection methods are frequently used in human ingestive behavior research (50). By applying visual-analogue scale questionnaires at the time of the drinkometer sessions, with the exception of postprandial nausea, we found no major differences between the groups despite the important changes observed in ingestive microstructure over time. Thus, the VAS scores used to assess stimulus liking, hunger, and thirst did not display a high predictive relationship for ingestive microstructure or even meal size.

Interestingly, the majority of patients after RYGB reported the desire of choosing a different flavor of the liquid meal, in the hypothetical scenario of being able to repeat the study from the beginning. In contrast, 90% of the participants of the control groups would have selected the same flavor again. This suggests that the flavor was associated with some aspect of the ingestive experience that increased its tendency to be avoided on further occasions. Although this could be considered in some ways a conditioned flavor aversion, the nature of the changes seen here relative to the healthy-weight control group suggest otherwise. As a caveat, the fact that this result was based on verbal report urges prudence in its interpretation with respect to the degree it would predict actual ingestive behavior.

An applied implication of our findings is related to the highly precise description of the postbariatric ingestive phenotype (51), which may provide a readily available reference for future weight loss therapies. In fact, a typical post-RYGB eating style may be characterized in future studies, including male participants and measurements of solid food intake. If such studies will confirm our findings, the pooled data may be used as a benchmark for behavioral interventions. Lack of self-control while eating is a well-described contributing factor of the obesity epidemic (52). Consequently, the cornerstone of cognitive interventions that prevent overeating and the risk of obesity is related to the deautomatization of eating habits (53). Undoubtedly, compliance represents the bottleneck of nonsurgical weight loss interventions and remains a vexing problem in medicine (54). An innovative way to assist patients in fulfilling nutritional recommendations is related to the application of wearable wireless devices and mobile phone applications. Preliminary experiences are promising: a wearable bite counter was effective in restricting portion size by helping participants to regulate their eating (52). In another example, experimenters used a meal-weighing device connected to a mobile application with real-time visual feedback on cumulative intake, which were shown to be successful in lowering BMI in obese children by training them to eat less and more slowly (55).

Another potential implication of ingestive behavioral analyses may be the optimization of the preoperative counseling process. Existing models of bariatric weight loss prediction tend to overestimate the outcome (56). However, a recent study published by Perez-Leighton et al. showed the utility of preoperative classification of patients according to their sucrose-wanting rating in the selection of BS type (57). Patients with a high-wanting profile lost more weight after RYGB in comparison with sleeve gastrectomy. In the mentioned study, patients were asked to test 3 sucrose and 3 aspartame concentrations in fasted condition and to report liking and wanting on VAS, which were used to clusterize participants. The particular efficacy of RYGB in patients with a preference for sweet foods has been confirmed in a study by Smith et al., where neural responses to varying concentrations of sucrose plus fat mixtures were captured by VAS and functional magnetic resonance imaging of the brain (58). Preoperative profiling of bariatric candidates in response to different liquid stimuli could be performed in the future by ingestive recordings. Ingestive microstructure of an entire meal could be assessed with a drinkometer or a sipometer. The sipometer has been recently validated to measure the desire to consume a particular tastant by inferring the reinforcing value of stimuli from the time and strengths exerted to sip them (59). Such technologies of direct measurements of ingestive behavior have the potential to overcome the constraints of self-reported taste preference assessments, but this comes at the price of robust logistical investments (hardware, software, consumables and manpower) and more time needed to collect and analyze data.

There are some limitations in our approach, which need to be considered when interpreting the data. First, the study was exploratory in nature, and our findings, which are based on a rather small cohort, need to be confirmed in larger groups of patients, with the use of solid food, and with the involvement of male participants. The viscosity/solidity of food has been shown to influence bite size in humans (28); therefore, the microstructure observed with liquid nutrients may not be representative for solid meals. Second, the interpretation of ingestive microstructure and its different parameters has been developed and validated in rodents with intact gastrointestinal anatomy. Human studies are scarce and have been mainly focused on the relationship of obesity and bite size, as well as deceleration of intake across a meal (60–63). Therefore, the physiologic readout and relevance of burst size and burst number — not only in humans, but also in the specific context of RYGB — warrant further investigations (64–66). The following factors may affect ingestive microstructure in humans and, thus, may have influenced our findings: sex (60), obesity (63), personal expectations on satiation (67), viscosity of the food (28), and the reward value of food (22). Third, even though we strove to include a homogenous cohort of female adults without significant comorbidities (nondiabetic, nonmedicated, nonpregnant, nonlactose intolerant, without major eating disorders), and despite the fact that we standardized environmental parameters (similar nutrient intake, minimal alcohol consumption, no sport activities preceding each measurement, and no access to food or drinks for 30 minutes after each session), we did not account for some additional baseline factors that could potentially influence ingestive behavior, such as cultural and social cues, idiosyncratic temperature preferences, and self-restraining factors (68). Fourth, the composition of the ingestive stimulus was constant across all measurements, which prevented the assessment of changes in relative macronutrient intake over time. This may be addressed in the future by providing participants the option of choosing between different stimuli presented from multiple reservoirs of the drinkometer.

In summary, we assessed the ingestive microstructure of a standardized liquid meal in the context of RYGB by a potentially novel and highly precise objective methodology. Significant differences in meal size between preoperative RYGB patients and healthy-weight controls vanished by the end of the first postoperative year. The post-RYGB meal size followed a dynamic adaptation process, with the highest decrease observed in the early postoperative phase. Specific microstructural elements of ingestion (especially burst size and meal duration) were found to account for the decreased meal intake after RYGB, suggesting that both increased postingestive sensibility and perhaps altered palatability may be mediators of the decreased ingestive behavior induced by RYGB. Microstructural analysis of eating and drinking still requires further validation in human physiological studies, and data should be complemented by 24-hour meal intake analyses, allowing the assessment of compensatory calorie intake and relative preferences of different macronutrients. Future studies should investigate the potential predictive significance of early changes in ingestive parameters and clinically relevant outcomes, such as postoperative weight loss. Nevertheless, our preliminary findings are relevant, since they (a) complement existing data regarding behavioral mechanisms implied in postbariatric changes of food preferences, (b) will assist the design of future studies by providing a range of expected ingestive values and microstructural parameters, and (c) may be used to enrich nutritional counseling by precise information on the bariatric patients’ ingestive phenotype.

## Methods

### Observational clinical study.

This was a prospective exploratory observational case-control study with 1-year follow-up performed at the University Hospital Zurich between 02/2018 and 01/2021. The manuscript was prepared according the “Strengthening the Reporting of Observational Studies in Epidemiology” guidelines for reporting observational studies (69).

### Participants.

The study group included 11 adult bariatric patients scheduled for RYGB for severe obesity, recruited preoperatively. The healthy-weight control group included 10 healthy young adults with a BMI between 18.5 and 25 kg/m^2^, and the control group of participants with severe obesity with no surgical or medical obesity treatment included 10 adults. The role of the control groups was to demonstrate test stability in these 2 distinct populations over time, since microstructural analysis of liquid meal intake has not been recorded previously in humans. The sample size was based on recent clinical and rodent studies that focused on ingestive behavior (33, 70), with the aim of generating data on the direction of changes in ingestive parameters based on the 136 meal sessions planned to be recorded in the study. To account for the potential bias in ingestive behavior related to sex, only female participants were included (44). Further exclusion criteria included severe eating disorder or pica, lactose-intolerance, diabetes mellitus, pregnancy/lactation, previous abdominal surgery, head and neck condition influencing meal intake, and inability to understand instructions in German, English, or French. To acknowledge their time and effort, participants received a compensation of 15 CHF per session.

### Ingestive stimulus.

A commercially available energy-dense standard liquid meal (Resource 2.0+Fibre, 2 kcal/mL, carbohydrate 43%, sugar 6.4%, fat 39%, protein 18%; gift from Nestlé Suisse S.A.) was chosen as ingestive stimulus to be consumed from the drinkometer. To account for idiosyncratic flavor preferences and aversions, participants had to choose 1 out of the 4 available flavors (chocolate, vanilla, strawberry, pineapple-mango). Once chosen, the selected flavor remained for each participant throughout all test sessions and was always served at refrigerated temperature (~6°C).

### Study procedure.

The RYGB patients performed 6 measurements: preoperative (1–2 weeks prior to RYGB) and 1 week postoperatively, as well as 1, 3, 6, and 12 months postoperatively. The healthy-weight control group was assessed in parallel at 4 time points: at baseline, and at 1, 3, and 12 months. The obese control group was recruited a posteriori to the previous 2 groups in order to solidify our findings, and it included 3 measurements: at baseline, and at 1 and 3 months. The technical aspects of the drinkometer were reported earlier by our group (44). In brief, the drinkometer constantly measures the weight of the fluid reservoir during the experiment. The acquisition of the weight sensor data is largely oversampled (1 kHz) to provide enough headroom for subsequent averaging filters (~10 Hz) to increase the signal/noise ratio and to avoid the noise generated by the first few harmonics of the power lines. An in-house–built postprocessing algorithm filters noise and identifies the beginning and the end of each suck by applying a multistep procedure based on the physiological and technical features of human drinking via the drinkometer. In the current study, participants were asked to consume a similar dinner and evening snack in terms of size, macronutrient composition, and total calories the night before each visit, and they were asked to avoid excessive alcohol consumption the evening before the test days, as well as to refrain from heavy exercise on the morning before testing. Prescribed and nonprescribed drugs were reported and documented. Body weight and height measurements were performed in light clothes and without shoes using calibrated scales and stadiometer. As sleep deprivation might increase hunger and thirst, we asked patients to report the amount of sleep they had the night before each ingestive session (71). Participants also reported their stage of female hormonal cycle, since food craving and macronutrient intake may be higher in the luteal phase (72). Participants were instructed to arrive around 2 p.m. after an overnight fast for food (solid and liquid) from 10 p.m. the previous day, but they were allowed to consume up to 1.5 L of water between 10 p.m. and 11 a.m. Participants were familiarized with the technical aspects of the drinkometer prior to the test sessions via a standardized individual 5-minute introduction by the same experimenter. Before each session, participants were told, “Consume until you feel full”. Drinking sessions were not limited in time; the 1 L capacity of the fluid reservoir represented the upper limit of the possible drinking volume. Pauses during drinking were allowed. The experimenter was hidden behind a separator curtain while the participants were consuming the liquid meal via a polyethylene tube. Participants were asked to rate their current level of hunger, thirst, and fullness immediately before and after, as well as 30 minutes after the drinking test on validated VAS (73). Extent of liking of the test solution, postprandial nausea, and abdominal pain were also captured. At the end of the study, participants were asked whether they would choose a different favor of the stimulus if they had to redo the study once again.

### Microstructural data acquisition and processing.

During each ingestive session, the drinkometer recorded the change of the meal volume on the scale of time, using the LabVIEW 2016 software (National Instruments). At the end of the study, all recordings were pooled and processed into an in-house–built algorithm (44) in Matlab R2016b software (MathWorks) for noise filtering and identification of sucks, bursts (i.e., group of sucks), and intervals separating them (ISI and IBI). First, the optimal burst PC was visualized and identified by PDF and Gaussian mixture models fitted to the frequency histograms log_e_ transformed ISI (49). Second, the identified PC was used to compute the microstructure of meal intake. Sucks can be visualized as complex waveforms representing the rate of fluid delivery into the mouth from the reservoir. Characteristics of the entire meal, as well as the number, size, duration, and rate of sucks, are PC independent, whereas the number, size, duration, rate of sucking bursts, and the lengths of within-meal pauses (ISI and IBI) are PC dependent.

### Statistics.

Data were analyzed for complete cases in R software version 4.0.4 (The R foundation for Statistical Computing). Normality of data distributions were assessed visually on Q-Q plots. To analyze the changes in recorded parameters from baseline, 2-way mixed ANOVA, with treatment group (RYGB or healthy-weight control) and postbaseline time points at which both groups were recorded (1 month, 3 month, 1 year) as factors were used on logarithmically transformed data reflecting proportion of baseline (log_10_
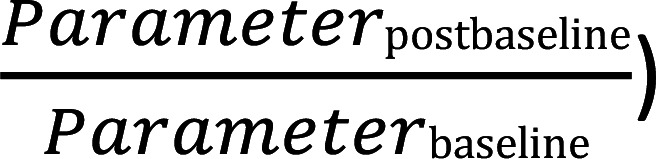
) (74). This information was designed so that no change would equal 0 and factor increases and decreases from baseline would be symmetrical (e.g., a 50% decrease, which equals a 2× reduction would equal a 200% increase, which equals a 2× elevation). Post hoc analyses were performed by Tukey’s honest significance test, and a *P* value of less than 0.05 was considered significant. The ingestive parameters showing a significant change in the RYGB versus healthy-weight groups were additionally tested with a 1-way ANOVA in the obese control group to confirm the stability of this parameters at repetitive encounters with the stimulus in the absence of bariatric surgery. Post hoc analyses were performed by Tukey’s honest significance test.

### Study approval.

The Cantonal Ethics Committee of Zurich approved this study (BASEC-Nr. 2017 — 00756). The experiments were carried out in accordance with The Code of Ethics of the World Medical Association (Declaration of Helsinki). The study was preregistered at ClinicalTrials.gov (NCT 03747445). All enrolled participants provided written informed consent for voluntary participation in the experiment and to deidentified use of their medical and physiologic records.

## Author contributions

MB, DG, ACS, and RES conceived the research question and designed the clinical study. DG conducted the clinical study, recruited patients, and recorded data. BF developed the data processing algorithm. DA and MS recorded part of data. AEMI critically reviewed the manuscript draft. DG, BF, LDF, and ACS performed data analysis. DG, MB, and ACS wrote the manuscript, and all authors contributed to the final version of the manuscript.

## Supplementary Material

Supplemental data

Trial reporting checklists

ICMJE disclosure forms

Supplemental video 1

## Figures and Tables

**Table 1:**
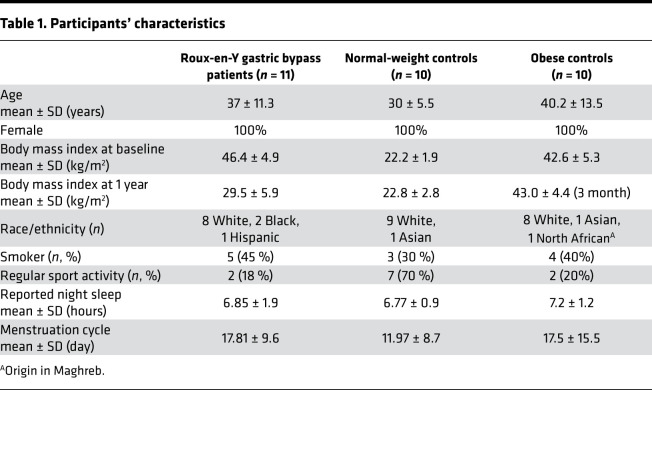
Participants’ characteristics

**Table 2:**
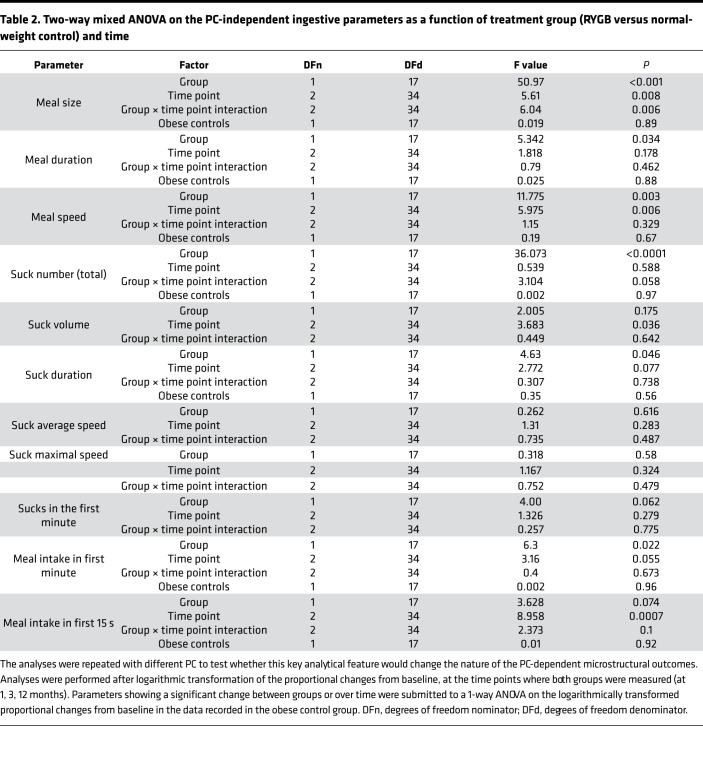
Two-way mixed ANOVA on the PC-independent ingestive parameters as a function of treatment group (RYGB versus normal-weight control) and time

**Table 3:**
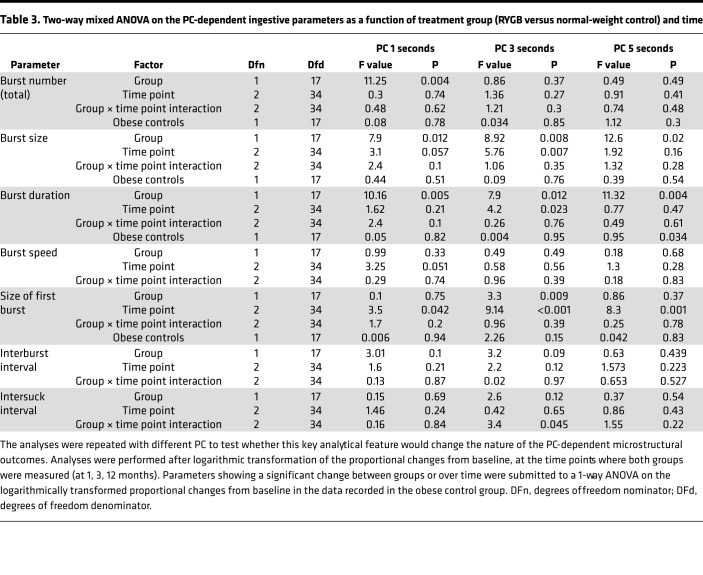
Two-way mixed ANOVA on the PC-dependent ingestive parameters as a function of treatment group (RYGB versus normal-weight control) and time

## Version 1 08/09/2021

Electronic publication
