# Supplementary material for: Microstructural changes in human ingestive behavior after Roux-en-Y gastric bypass during liquid meals
Source: JCI Insight. 2021 Aug 9;6(15):e136842. doi: 10.1172/jci.insight.136842 (PMC8410040; doi:10.1172/jci.insight.136842)

Supplemental Digital Content

Microstructural changes in human ingestive behavior after Roux-en-Y gastric bypass during liquid meals. *Gero et al.* JCI Insight 2021

Supplemental Figure 1. Overall ingestive parameters over time (group means: bold lines, individual curves: pale lines). A. Calorie intake (1 ml of the stimulus contained 2 kCal); B. Meal duration; C. Average drinking speed, D. Total number of sucks. *Blue: Roux-en-Y gastric bypass group; Red: Normal-weight control group; Green: Obese control group.*

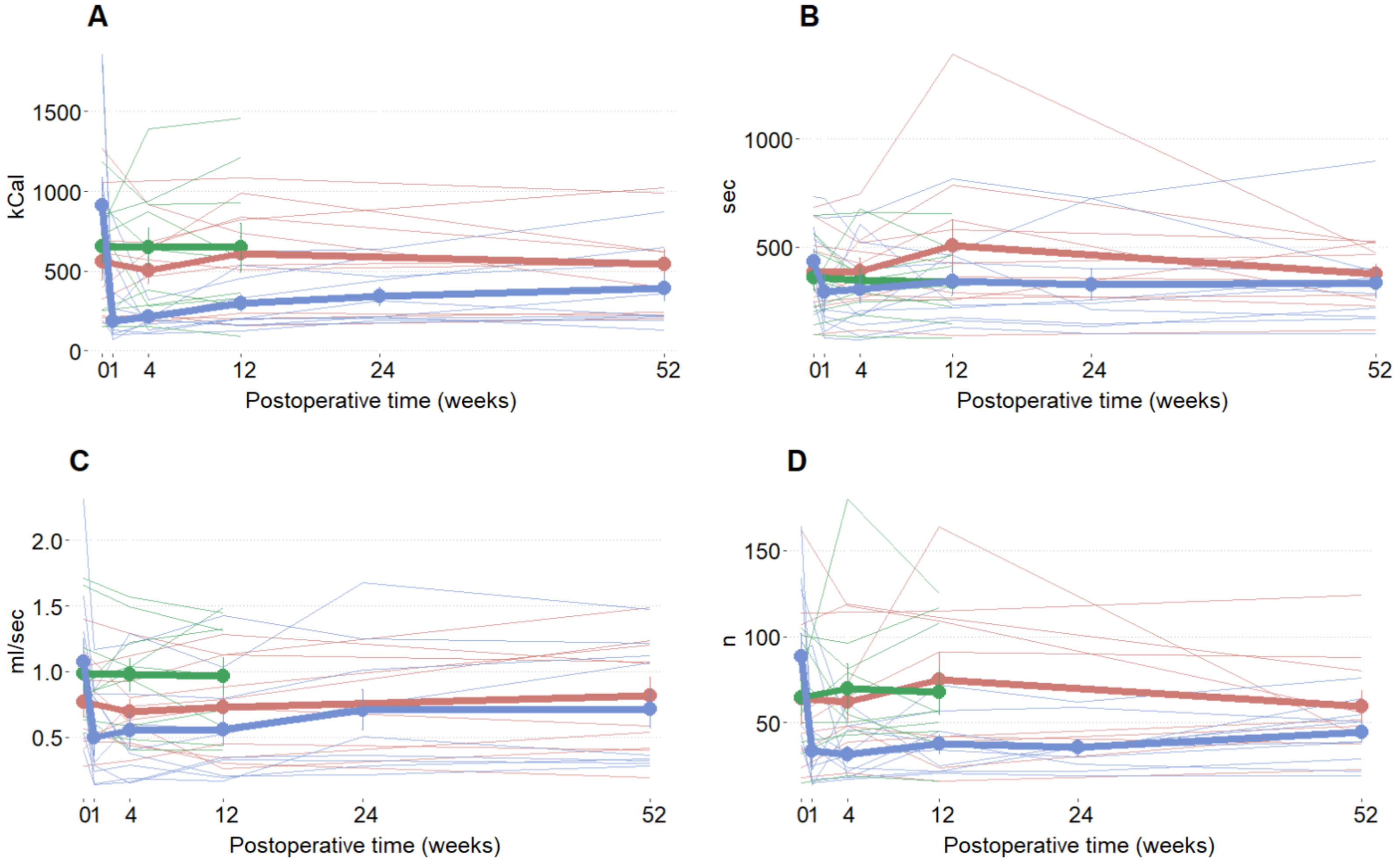

Supplemental Figure 2. Microstructural parameters over time (group means: bold lines, individual curves: pale lines; burst-pause criterion = 3 s). A. Suck volume; B. Mean burst size; C. Total number of bursts; D. Inter-burst interval. *Blue: Roux-en-Y gastric bypass group; Red: Normal-weight control group; Green: Obese control group.*

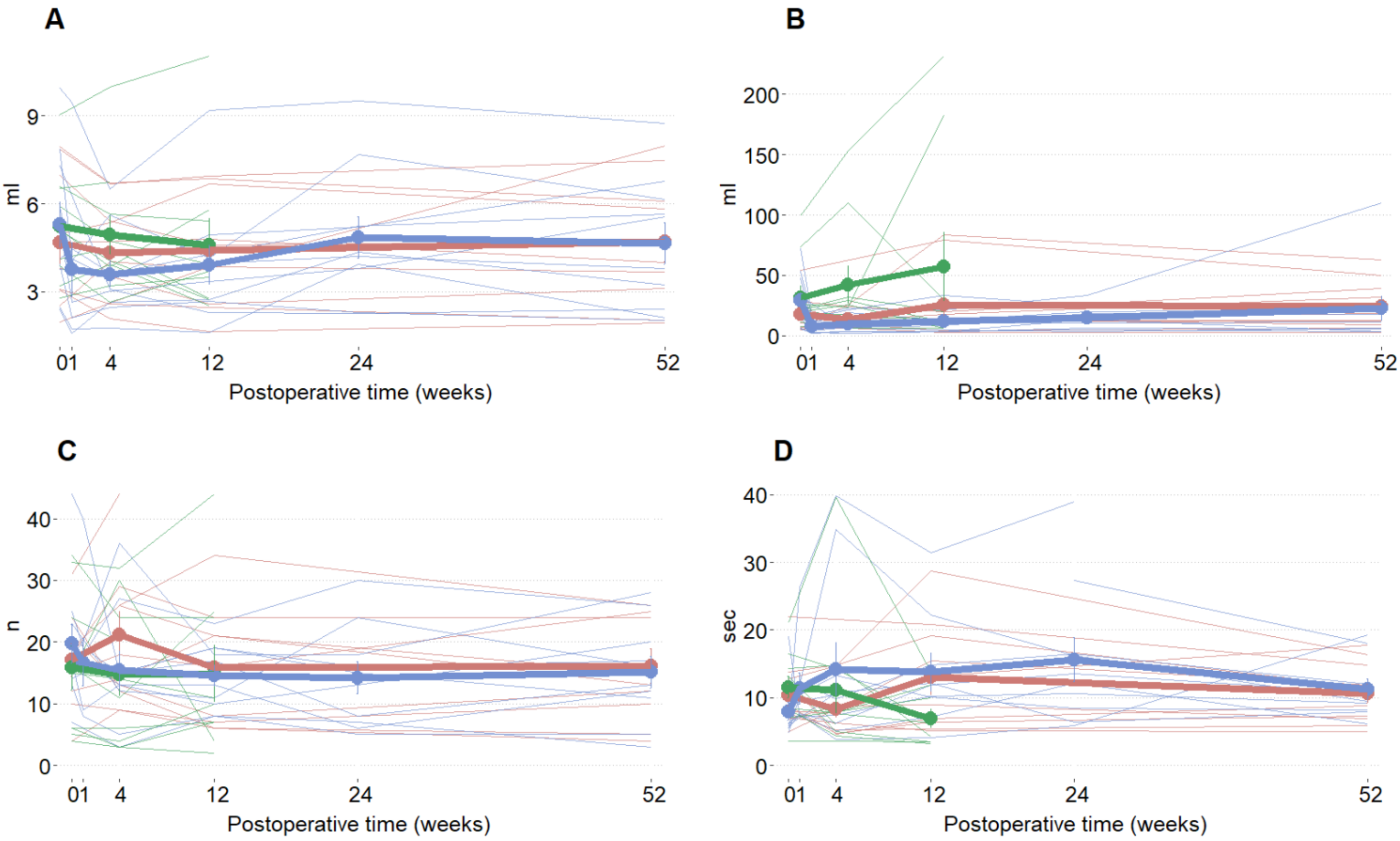

Supplemental Figure 3. Microstructural parameters at the beginning of the meal (group means: bold lines, individual curves: pale lines). A. Size of the first burst (PC = 3 s); B. Intake in the first 15 s; C. Intake in the first 60 s; D. Number of sucks within the first minute. *Blue: Roux-en-Y gastric bypass group; Red: Normal-weight control group; Green: Obese control group.*

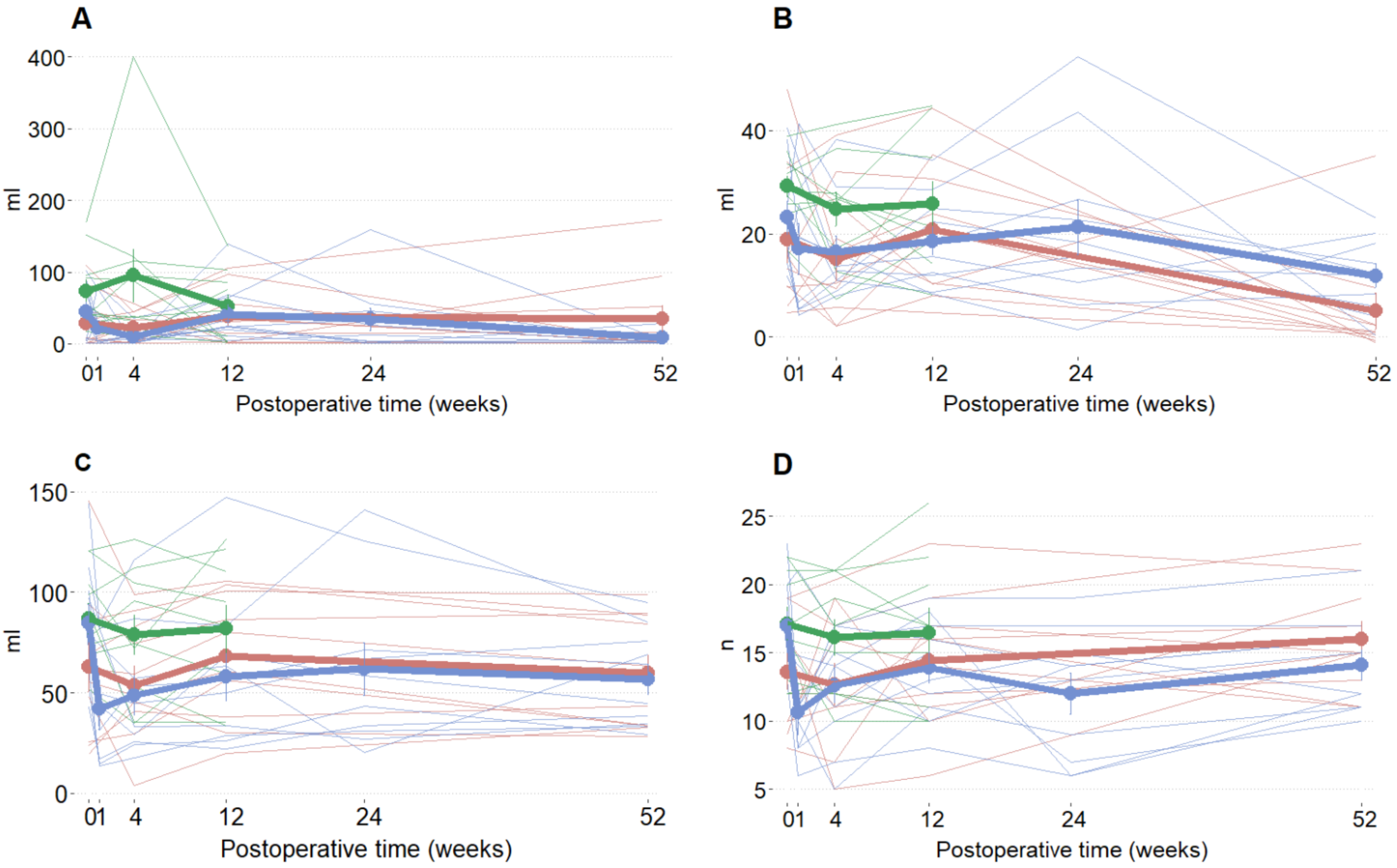

Supplement: Supplemental data [file jciinsight-6-136842-s260.pdf]
