# Supplementary material for: Microstructural changes in human ingestive behavior after Roux-en-Y gastric bypass during liquid meals
Source: JCI Insight. 2021 Aug 9;6(15):e136842. doi: 10.1172/jci.insight.136842 (PMC8410040; doi:10.1172/jci.insight.136842)
Supplement: ICMJE disclosure forms [file jciinsight-6-136842-s262.pdf]

## ICMJE DISCLOSURE FORM

**Date:_____________March 15,2021___________________________________________________**
**Your Name:_____Daniel Gero_________________________________________________________________________**
**Manuscript Title:** Microstructural changes in human ingestive behavior after Roux-en-Y gastric bypass during liquid meals
**Manuscript number (if known):** Reference: 136842-INS-CMED-TR-2

**In the interest of transparency, we ask you to disclose all relationships/activities/interests listed below that are related to the content of your manuscript. "Related" means any relation with for-profit or not-for-profit third parties whose interests may be affected by the content of the manuscript. Disclosure represents a commitment to transparency and does not necessarily indicate a bias. If you are in doubt about whether to list a relationship/activity/interest, it is preferable that you do so.**

**The following questions apply to the author's relationships/activities/interests as they relate to the current manuscript only.**

**The author's relationships/activities/interests should be defined broadly. For example, if your manuscript pertains to the epidemiology of hypertension, you should declare all relationships with manufacturers of antihypertensive medication, even if that medication is not mentioned in the manuscript.**

**In item #1 below, report all support for the work reported in this manuscript without time limit. For all other items, the time frame for disclosure is the past 36 months.**

| | | Name all entities with whom you have this relationship or indicate none (add rows as needed) | Specifications/Comments (e.g., if payments were made to you or to your institution) |
|---|---|---|---|
| | | **Time frame: Since the initial planning of the work** | |
| 1 | All support for the present manuscript (e.g., funding, provision of study materials, medical writing, article processing charges, etc.) **No time limit for this item.** | ____None | |
| | | | |
| | | | |
| | | | |
| | | | |
| | | | |
| | | | |
| | | | |
| | | **Time frame: past 36 months** | |
| 2 | Grants or contracts from any entity (if not indicated in item #1 above). | ____None | |
| | | | |
| | | | |
| 3 | Royalties or licenses | ____None | |
| | | | |
| | | | |
| 4 | Consulting fees | ____None | |
| | | | |

| | | | |
|---|---|---|---|
| 5 | Payment or honoraria for lectures, presentations, speakers bureaus, manuscript writing or educational events | ____None | |
| | | | |
| | | | |
| 6 | Payment for expert testimony | ____None | |
| | | | |
| | | | |
| 7 | Support for attending meetings and/or travel | ____None | |
| | | | |
| | | | |
| 8 | Patents planned, issued or pending | ____None | |
| | | | |
| | | | |
| 9 | Participation on a Data Safety Monitoring Board or Advisory Board | ____None | |
| | | | |
| | | | |
| 10 | Leadership or fiduciary role in other board, society, committee or advocacy group, paid or unpaid | ____None | |
| | | | |
| | | | |
| 11 | Stock or stock options | ____None | |
| | | | |
| | | | |
| 12 | Receipt of equipment, materials, drugs, medical writing, gifts or other services | ____None | |
| | | | |
| | | | |
| 13 | Other financial or non-financial interests | ____None | |
| | | | |
| | | | |

**Please place an "X" next to the following statement to indicate your agreement:**

**_X_ I certify that I have answered every question and have not altered the wording of any of the questions on this form.**

## ICMJE DISCLOSURE FORM

**Date:______________March 15,2021________________________________________________________**
**Your Name:_________Bálint File___________________________________________________**

**Manuscript Title:** Microstructural changes in human ingestive behavior after Roux-en-Y gastric bypass during liquid meals

**Manuscript number (if known):** Reference: 136842-INS-CMED-TR-2

**In the interest of transparency, we ask you to disclose all relationships/activities/interests listed below that are related to the content of your manuscript. "Related" means any relation with for-profit or not-for-profit third parties whose interests may be affected by the content of the manuscript. Disclosure represents a commitment to transparency and does not necessarily indicate a bias. If you are in doubt about whether to list a relationship/activity/interest, it is preferable that you do so.**

**The following questions apply to the author's relationships/activities/interests as they relate to the current manuscript only.**

**The author's relationships/activities/interests should be defined broadly. For example, if your manuscript pertains to the epidemiology of hypertension, you should declare all relationships with manufacturers of antihypertensive medication, even if that medication is not mentioned in the manuscript.**

**In item #1 below, report all support for the work reported in this manuscript without time limit. For all other items, the time frame for disclosure is the past 36 months.**

| | | **Name all entities with whom you have this relationship or indicate none (add rows as needed)** | **Specifications/Comments (e.g., if payments were made to you or to your institution)** |
|---|---|---|---|
| **Time frame: Since the initial planning of the work** | | | |
| 1 | All support for the present manuscript (e.g., funding, provision of study materials, medical writing, article processing charges, etc.) **No time limit for this item.** | Hungarian Brain Research Program Grant (Grant No. 2017-1.2.1-NKP-2017-00002) | |
| | | | |
| | | | |
| | | | |
| | | | |
| | | | |
| | | | |
| | | | |
| **Time frame: past 36 months** | | | |
| 2 | Grants or contracts from any entity (if not indicated in item #1 above). | ____None | |
| | | | |
| | | | |

| | | | |
|---|---|---|---|
| 3 | Royalties or licenses | ____None | |
| | | | |
| | | | |
| 4 | Consulting fees | ____None | |
| | | | |
| | | | |
| 5 | Payment or honoraria for lectures, presentations, speakers bureaus, manuscript writing or educational events | ____None | |
| | | | |
| | | | |
| 6 | Payment for expert testimony | ____None | |
| | | | |
| | | | |
| 7 | Support for attending meetings and/or travel | ____None | |
| | | | |
| | | | |
| 8 | Patents planned, issued or pending | ____None | |
| | | | |
| | | | |
| 9 | Participation on a Data Safety Monitoring Board or Advisory Board | ____None | |
| | | | |
| | | | |
| 10 | Leadership or fiduciary role in other board, society, committee or advocacy group, paid or unpaid | ____None | |
| | | | |
| | | | |
| 11 | Stock or stock options | ____None | |
| | | | |
| | | | |
| 12 | Receipt of equipment, materials, drugs, medical writing, gifts or other services | ____None | |
| | | | |
| | | | |
| 13 | Other financial or non-financial interests | ____None | |
| | | | |
| | | | |

**Please place an "X" next to the following statement to indicate your agreement:**

**_X__ I certify that I have answered every question and have not altered the wording of any of the questions on this form.**

## ICMJE DISCLOSURE FORM

**Date:**_____________**March 15,2021**___________________________________________________
**Your Name:**_________**Daniela Alceste**______________________________________________________________
**Manuscript Title:** Microstructural changes in human ingestive behavior after Roux-en-Y gastric bypass during liquid meals
**Manuscript number (if known):** Reference: 136842-INS-CMED-TR-2

**In the interest of transparency, we ask you to disclose all relationships/activities/interests listed below that are related to the content of your manuscript. "Related" means any relation with for-profit or not-for-profit third parties whose interests may be affected by the content of the manuscript. Disclosure represents a commitment to transparency and does not necessarily indicate a bias. If you are in doubt about whether to list a relationship/activity/interest, it is preferable that you do so.**

**The following questions apply to the author's relationships/activities/interests as they relate to the current manuscript only.**

**The author's relationships/activities/interests should be defined broadly. For example, if your manuscript pertains to the epidemiology of hypertension, you should declare all relationships with manufacturers of antihypertensive medication, even if that medication is not mentioned in the manuscript.**

**In item #1 below, report all support for the work reported in this manuscript without time limit. For all other items, the time frame for disclosure is the past 36 months.**

| | | **Name all entities with whom you have this relationship or indicate none (add rows as needed)** | **Specifications/Comments (e.g., if payments were made to you or to your institution)** |
|---|---|---|---|
| | | **Time frame: Since the initial planning of the work** | |
| 1 | All support for the present manuscript (e.g., funding, provision of study materials, medical writing, article processing charges, etc.) **No time limit for this item.** | ____None | |
| | | | |
| | | | |
| | | | |
| | | | |
| | | | |
| | | | |
| | | | |
| | | **Time frame: past 36 months** | |
| 2 | Grants or contracts from any entity (if not indicated in item #1 above). | ____None | |
| | | | |
| | | | |
| 3 | Royalties or licenses | ____None | |
| | | | |
| | | | |
| 4 | Consulting fees | ____None | |
| | | | |

| | | | |
|---|---|---|---|
| 5 | Payment or honoraria for lectures, presentations, speakers bureaus, manuscript writing or educational events | ____None | |
| | | | |
| | | | |
| 6 | Payment for expert testimony | ____None | |
| | | | |
| | | | |
| 7 | Support for attending meetings and/or travel | ____None | |
| | | | |
| | | | |
| 8 | Patents planned, issued or pending | ____None | |
| | | | |
| | | | |
| 9 | Participation on a Data Safety Monitoring Board or Advisory Board | ____None | |
| | | | |
| | | | |
| 10 | Leadership or fiduciary role in other board, society, committee or advocacy group, paid or unpaid | ____None | |
| | | | |
| | | | |
| 11 | Stock or stock options | ____None | |
| | | | |
| | | | |
| 12 | Receipt of equipment, materials, drugs, medical writing, gifts or other services | ____None | |
| | | | |
| | | | |
| 13 | Other financial or non-financial interests | ____None | |
| | | | |
| | | | |

**Please place an "X" next to the following statement to indicate your agreement:**

**__X_ I certify that I have answered every question and have not altered the wording of any of the questions on this form.**

## ICMJE DISCLOSURE FORM

**Date:_____________March 15,2021_________________________________________________**
**Your Name:_______Lukas Frick_____________________________________________________**
**Manuscript Title:** Microstructural changes in human ingestive behavior after Roux-en-Y gastric bypass during liquid meals
**Manuscript number (if known):** Reference: 136842-INS-CMED-TR-2

**In the interest of transparency, we ask you to disclose all relationships/activities/interests listed below that are related to the content of your manuscript. "Related" means any relation with for-profit or not-for-profit third parties whose interests may be affected by the content of the manuscript. Disclosure represents a commitment to transparency and does not necessarily indicate a bias. If you are in doubt about whether to list a relationship/activity/interest, it is preferable that you do so.**

**The following questions apply to the author's relationships/activities/interests as they relate to the current manuscript only.**

**The author's relationships/activities/interests should be defined broadly. For example, if your manuscript pertains to the epidemiology of hypertension, you should declare all relationships with manufacturers of antihypertensive medication, even if that medication is not mentioned in the manuscript.**

**In item #1 below, report all support for the work reported in this manuscript without time limit. For all other items, the time frame for disclosure is the past 36 months.**

| | | **Name all entities with whom you have this relationship or indicate none (add rows as needed)** | **Specifications/Comments (e.g., if payments were made to you or to your institution)** |
|---|---|---|---|
| | | **Time frame: Since the initial planning of the work** | |
| 1 | All support for the present manuscript (e.g., funding, provision of study materials, medical writing, article processing charges, etc.) **No time limit for this item.** | ____None | |
| | | | |
| | | | |
| | | | |
| | | | |
| | | | |
| | | | |
| | | | |
| | | **Time frame: past 36 months** | |
| 2 | Grants or contracts from any entity (if not indicated in item #1 above). | ____None | |
| | | | |
| | | | |
| 3 | Royalties or licenses | ____None | |
| | | | |
| | | | |
| 4 | Consulting fees | ____None | |
| | | | |

| | | | |
|---|---|---|---|
| 5 | Payment or honoraria for lectures, presentations, speakers bureaus, manuscript writing or educational events | ____None | |
| | | | |
| | | | |
| 6 | Payment for expert testimony | ____None | |
| | | | |
| | | | |
| 7 | Support for attending meetings and/or travel | ____None | |
| | | | |
| | | | |
| 8 | Patents planned, issued or pending | ____None | |
| | | | |
| | | | |
| 9 | Participation on a Data Safety Monitoring Board or Advisory Board | ____None | |
| | | | |
| | | | |
| 10 | Leadership or fiduciary role in other board, society, committee or advocacy group, paid or unpaid | ____None | |
| | | | |
| | | | |
| 11 | Stock or stock options | ____None | |
| | | | |
| | | | |
| 12 | Receipt of equipment, materials, drugs, medical writing, gifts or other services | ____None | |
| | | | |
| | | | |
| 13 | Other financial or non-financial interests | ____None | |
| | | | |
| | | | |

**Please place an "X" next to the following statement to indicate your agreement:**

**__X_ I certify that I have answered every question and have not altered the wording of any of the questions on this form.**

ICMJE DISCLOSURE FORM

**Date:_____________March 15,2021_____________________________________________________**
**Your Name:______Michele Serra_____________________________________________________**
**Manuscript Title:** Microstructural changes in human ingestive behavior after Roux-en-Y gastric bypass during liquid meals
**Manuscript number (if known):** Reference: 136842-INS-CMED-TR-2

**In the interest of transparency, we ask you to disclose all relationships/activities/interests listed below that are related to the content of your manuscript. "Related" means any relation with for-profit or not-for-profit third parties whose interests may be affected by the content of the manuscript. Disclosure represents a commitment to transparency and does not necessarily indicate a bias. If you are in doubt about whether to list a relationship/activity/interest, it is preferable that you do so.**

**The following questions apply to the author's relationships/activities/interests as they relate to the current manuscript only.**

**The author's relationships/activities/interests should be defined broadly. For example, if your manuscript pertains to the epidemiology of hypertension, you should declare all relationships with manufacturers of antihypertensive medication, even if that medication is not mentioned in the manuscript.**

**In item #1 below, report all support for the work reported in this manuscript without time limit. For all other items, the time frame for disclosure is the past 36 months.**

| | | **Name all entities with whom you have this relationship or indicate none (add rows as needed)** | **Specifications/Comments (e.g., if payments were made to you or to your institution)** |
|---|---|---|---|
| | | **Time frame: Since the initial planning of the work** | |
| 1 | All support for the present manuscript (e.g., funding, provision of study materials, medical writing, article processing charges, etc.) **No time limit for this item.** | ____None | |
| | | | |
| | | | |
| | | | |
| | | | |
| | | | |
| | | | |
| | | | |
| | | **Time frame: past 36 months** | |
| 2 | Grants or contracts from any entity (if not indicated in item #1 above). | ____None | |
| | | | |
| | | | |
| 3 | Royalties or licenses | ____None | |
| | | | |
| | | | |
| 4 | Consulting fees | ____None | |
| | | | |

| | | | |
|---|---|---|---|
| 5 | Payment or honoraria for lectures, presentations, speakers bureaus, manuscript writing or educational events | ____None | |
| | | | |
| | | | |
| 6 | Payment for expert testimony | ____None | |
| | | | |
| | | | |
| 7 | Support for attending meetings and/or travel | ____None | |
| | | | |
| | | | |
| 8 | Patents planned, issued or pending | ____None | |
| | | | |
| | | | |
| 9 | Participation on a Data Safety Monitoring Board or Advisory Board | ____None | |
| | | | |
| | | | |
| 10 | Leadership or fiduciary role in other board, society, committee or advocacy group, paid or unpaid | ____None | |
| | | | |
| | | | |
| 11 | Stock or stock options | ____None | |
| | | | |
| | | | |
| 12 | Receipt of equipment, materials, drugs, medical writing, gifts or other services | ____None | |
| | | | |
| | | | |
| 13 | Other financial or non-financial interests | ____None | |
| | | | |
| | | | |

**Please place an "X" next to the following statement to indicate your agreement:**

**__X_ I certify that I have answered every question and have not altered the wording of any of the questions on this form.**

## ICMJE DISCLOSURE FORM

**Date:**_____________**March 15,2021**______________________________________________________
**Your Name:**______ Aiman E. M. Ismaeil ________________________________________
**Manuscript Title:** Microstructural changes in human ingestive behavior after Roux-en-Y gastric bypass during liquid meals
**Manuscript number (if known):** Reference: 136842-INS-CMED-TR-2

**In the interest of transparency, we ask you to disclose all relationships/activities/interests listed below that are related to the content of your manuscript. "Related" means any relation with for-profit or not-for-profit third parties whose interests may be affected by the content of the manuscript. Disclosure represents a commitment to transparency and does not necessarily indicate a bias. If you are in doubt about whether to list a relationship/activity/interest, it is preferable that you do so.**

**The following questions apply to the author's relationships/activities/interests as they relate to the current manuscript only.**

**The author's relationships/activities/interests should be defined broadly. For example, if your manuscript pertains to the epidemiology of hypertension, you should declare all relationships with manufacturers of antihypertensive medication, even if that medication is not mentioned in the manuscript.**

**In item #1 below, report all support for the work reported in this manuscript without time limit. For all other items, the time frame for disclosure is the past 36 months.**

| | | **Name all entities with whom you have this relationship or indicate none (add rows as needed)** | **Specifications/Comments (e.g., if payments were made to you or to your institution)** |
|---|---|---|---|
| | | **Time frame: Since the initial planning of the work** | |
| 1 | All support for the present manuscript (e.g., funding, provision of study materials, medical writing, article processing charges, etc.) **No time limit for this item.** | ____None | |
| | | | |
| | | | |
| | | | |
| | | | |
| | | | |
| | | | |
| | | | |
| | | **Time frame: past 36 months** | |
| 2 | Grants or contracts from any entity (if not indicated in item #1 above). | ____None | |
| | | | |
| | | | |
| 3 | Royalties or licenses | ____None | |
| | | | |
| | | | |
| 4 | Consulting fees | ____None | |
| | | | |

| | | | |
|---|---|---|---|
| 5 | Payment or honoraria for lectures, presentations, speakers bureaus, manuscript writing or educational events | ____None | |
| | | | |
| | | | |
| 6 | Payment for expert testimony | ____None | |
| | | | |
| | | | |
| 7 | Support for attending meetings and/or travel | ____None | |
| | | | |
| | | | |
| 8 | Patents planned, issued or pending | ____None | |
| | | | |
| | | | |
| 9 | Participation on a Data Safety Monitoring Board or Advisory Board | ____None | |
| | | | |
| | | | |
| 10 | Leadership or fiduciary role in other board, society, committee or advocacy group, paid or unpaid | ____None | |
| | | | |
| | | | |
| 11 | Stock or stock options | ____None | |
| | | | |
| | | | |
| 12 | Receipt of equipment, materials, drugs, medical writing, gifts or other services | ____None | |
| | | | |
| | | | |
| 13 | Other financial or non-financial interests | ____None | |
| | | | |
| | | | |

**Please place an "X" next to the following statement to indicate your agreement:**

**_X__ I certify that I have answered every question and have not altered the wording of any of the questions on this form.**

## ICMJE DISCLOSURE FORM

**Date:_____________March 15,2021_____________________________________________________**
**Your Name:______** Robert E Steinert _______________________________________
**Manuscript Title:** Microstructural changes in human ingestive behavior after Roux-en-Y gastric bypass during liquid meals
**Manuscript number (if known):** Reference: 136842-INS-CMED-TR-2

**In the interest of transparency, we ask you to disclose all relationships/activities/interests listed below that are related to the content of your manuscript. "Related" means any relation with for-profit or not-for-profit third parties whose interests may be affected by the content of the manuscript. Disclosure represents a commitment to transparency and does not necessarily indicate a bias. If you are in doubt about whether to list a relationship/activity/interest, it is preferable that you do so.**

**The following questions apply to the author's relationships/activities/interests as they relate to the current manuscript only.**

**The author's relationships/activities/interests should be defined broadly. For example, if your manuscript pertains to the epidemiology of hypertension, you should declare all relationships with manufacturers of antihypertensive medication, even if that medication is not mentioned in the manuscript.**

**In item #1 below, report all support for the work reported in this manuscript without time limit. For all other items, the time frame for disclosure is the past 36 months.**

| | | **Name all entities with whom you have this relationship or indicate none (add rows as needed)** | **Specifications/Comments (e.g., if payments were made to you or to your institution)** |
|---|---|---|---|
| | | **Time frame: Since the initial planning of the work** | |
| 1 | All support for the present manuscript (e.g., funding, provision of study materials, medical writing, article processing charges, etc.) **No time limit for this item.** | ____None | |
| | | | |
| | | | |
| | | | |
| | | | |
| | | | |
| | | | |
| | | | |
| | | **Time frame: past 36 months** | |
| 2 | Grants or contracts from any entity (if not indicated in item #1 above). | Olga Mayenfisch Foundation | |
| | | | |
| | | | |
| 3 | Royalties or licenses | ____None | |
| | | | |
| | | | |

| | | | |
|---|---|---|---|
| 4 | Consulting fees | ____None | |
| | | | |
| | | | |
| 5 | Payment or honoraria for lectures, presentations, speakers bureaus, manuscript writing or educational events | ____None | |
| | | | |
| | | | |
| 6 | Payment for expert testimony | ____None | |
| | | | |
| | | | |
| 7 | Support for attending meetings and/or travel | ____None | |
| | | | |
| | | | |
| 8 | Patents planned, issued or pending | ____None | |
| | | | |
| | | | |
| 9 | Participation on a Data Safety Monitoring Board or Advisory Board | ____None | |
| | | | |
| | | | |
| 10 | Leadership or fiduciary role in other board, society, committee or advocacy group, paid or unpaid | ____None | |
| | | | |
| | | | |
| 11 | Stock or stock options | ____None | |
| | | | |
| | | | |
| 12 | Receipt of equipment, materials, drugs, medical writing, gifts or other services | ____None | |
| | | | |
| | | | |
| 13 | Other financial or non-financial interests | _Employed by D.S.M Nutritional Products | |
| | | | |
| | | | |

**Please place an "X" next to the following statement to indicate your agreement:**

**_X__ I certify that I have answered every question and have not altered the wording of any of the questions on this form.**

## ICMJE DISCLOSURE FORM

**Date:_____________March 15,2021_____________________________________________________**
**Your Name:______** Alan C Spector **_______________________________________**
**Manuscript Title:** Microstructural changes in human ingestive behavior after Roux-en-Y gastric bypass during liquid meals
**Manuscript number (if known):** Reference: 136842-INS-CMED-TR-2

**In the interest of transparency, we ask you to disclose all relationships/activities/interests listed below that are related to the content of your manuscript. "Related" means any relation with for-profit or not-for-profit third parties whose interests may be affected by the content of the manuscript. Disclosure represents a commitment to transparency and does not necessarily indicate a bias. If you are in doubt about whether to list a relationship/activity/interest, it is preferable that you do so.**

**The following questions apply to the author's relationships/activities/interests as they relate to the current manuscript only.**

**The author's relationships/activities/interests should be defined broadly. For example, if your manuscript pertains to the epidemiology of hypertension, you should declare all relationships with manufacturers of antihypertensive medication, even if that medication is not mentioned in the manuscript.**

**In item #1 below, report all support for the work reported in this manuscript without time limit. For all other items, the time frame for disclosure is the past 36 months.**

| | | **Name all entities with whom you have this relationship or indicate none (add rows as needed)** | **Specifications/Comments (e.g., if payments were made to you or to your institution)** |
|---|---|---|---|
| | | **Time frame: Since the initial planning of the work** | |
| 1 | All support for the present manuscript (e.g., funding, provision of study materials, medical writing, article processing charges, etc.) **No time limit for this item.** | ____None | |
| | | | |
| | | | |
| | | | |
| | | | |
| | | | |
| | | | |
| | | | |
| | | **Time frame: past 36 months** | |
| 2 | Grants or contracts from any entity (if not indicated in item #1 above). | ____None | |
| | | | |
| | | | |
| 3 | Royalties or licenses | ____None | |
| | | | |
| | | | |
| 4 | Consulting fees | ____None | |
| | | | |

| | | | |
|---|---|---|---|
| 5 | Payment or honoraria for lectures, presentations, speakers bureaus, manuscript writing or educational events | 23 March 2018: Interdisciplinary Mind and Brain Seminar, University of Pennsylvania, Philadelphia, PA.<br><br>27 March 2018: Neuroscience Program, Beckman Institute, University of Illinois, Urbana-Champagne, IL<br><br>14 May 2018: Bohan Seminar, University of Kansas Medical Center, Kansas City, KS.<br><br>12 November 2018: Symposium, Pennington Research Center, LSU, Baton Rouge, LA.<br><br>12 November 2020: Symposium , Neuroscience Institute, University of Texas San Antonio | Received honoraria for talks on experimental work conducted in my laboratory studying the effect of RYGB on taste and feeding in rodents. |
| | | | |
| | | | |
| 6 | Payment for expert testimony | ____None | |
| | | | |
| | | | |
| 7 | Support for attending meetings and/or travel | 2018: Neuroscience Program, Beckman Institute, University of Illinois, Urbana-Champagne, IL<br><br>14 May 2018: Bohan Seminar, University of Kansas Medical Center, Kansas City, KS.<br><br>4 December 2018: Symposium, Pennington Research Center, LSU, Baton Rouge, LA. | |
| | | | |
| | | | |

| | | | |
|---|---|---|---|
| 8 | Patents planned, issued or pending | ____None | |
| | | | |
| | | | |
| 9 | Participation on a Data Safety Monitoring Board or Advisory Board | ____None | |
| | | | |
| | | | |
| 10 | Leadership or fiduciary role in other board, society, committee or advocacy group, paid or unpaid | ____None | |
| | | | |
| | | | |
| 11 | Stock or stock options | ____None | |
| | | | |
| | | | |
| 12 | Receipt of equipment, materials, drugs, medical writing, gifts or other services | ____None | |
| | | | |
| | | | |
| 13 | Other financial or non-financial interests | ____None | |
| | | | |
| | | | |

**Please place an "X" next to the following statement to indicate your agreement:**

**_X__ I certify that I have answered every question and have not altered the wording of any of the questions on this form.**

## ICMJE DISCLOSURE FORM

**Date:**_____________**March 15,2021**_____________________________________________________

**Your Name:**______ Marco Bueter _______________________________________

**Manuscript Title:** Microstructural changes in human ingestive behavior after Roux-en-Y gastric bypass during liquid meals

**Manuscript number (if known):** Reference: 136842-INS-CMED-TR-2

**In the interest of transparency, we ask you to disclose all relationships/activities/interests listed below that are related to the content of your manuscript. "Related" means any relation with for-profit or not-for-profit third parties whose interests may be affected by the content of the manuscript. Disclosure represents a commitment to transparency and does not necessarily indicate a bias. If you are in doubt about whether to list a relationship/activity/interest, it is preferable that you do so.**

**The following questions apply to the author's relationships/activities/interests as they relate to the current manuscript only.**

**The author's relationships/activities/interests should be defined broadly. For example, if your manuscript pertains to the epidemiology of hypertension, you should declare all relationships with manufacturers of antihypertensive medication, even if that medication is not mentioned in the manuscript.**

**In item #1 below, report all support for the work reported in this manuscript without time limit. For all other items, the time frame for disclosure is the past 36 months.**

| | | **Name all entities with whom you have this relationship or indicate none (add rows as needed)** | **Specifications/Comments (e.g., if payments were made to you or to your institution)** |
|---|---|---|---|
| **Time frame: Since the initial planning of the work** | | | |
| 1 | All support for the present manuscript (e.g., funding, provision of study materials, medical writing, article processing charges, etc.) **No time limit for this item.** | University of Zurich | Assistant Professorship Grant |
| | | | |
| | | | |
| | | | |
| | | | |
| | | | |
| | | | |
| | | | |
| **Time frame: past 36 months** | | | |
| 2 | Grants or contracts from any entity (if not indicated in item #1 above). | Swiss National Science Foundation | Grant number 32003B_182309 |
| | | Olga Mayenfisch Foundation | |
| | | | |
| 3 | Royalties or licenses | ____None | |
| | | | |

| | | | |
|---|---|---|---|
| 4 | Consulting fees | ____None | |
| | | | |
| | | | |
| 5 | Payment or honoraria for lectures, presentations, speakers bureaus, manuscript writing or educational events | Johnson & Johnson | |
| | | Medtronic | |
| | | | |
| 6 | Payment for expert testimony | ____None | |
| | | | |
| | | | |
| 7 | Support for attending meetings and/or travel | ____None | |
| | | | |
| | | | |
| 8 | Patents planned, issued or pending | ____None | |
| | | | |
| | | | |
| 9 | Participation on a Data Safety Monitoring Board or Advisory Board | ____None | |
| | | | |
| | | | |
| 10 | Leadership or fiduciary role in other board, society, committee or advocacy group, paid or unpaid | Swiss Society for the Study of Obesity and Metabolic Diseases (SMOB) | Vice President 2017 - 2021 |
| | | | |
| | | | |
| 11 | Stock or stock options | ____None | |
| | | | |
| | | | |
| 12 | Receipt of equipment, materials, drugs, medical writing, gifts or other services | ____None | |
| | | | |
| | | | |
| 13 | Other financial or non-financial interests | ____None | |
| | | | |
| | | | |

**Please place an "X" next to the following statement to indicate your agreement:**

**_X__ I certify that I have answered every question and have not altered the wording of any of the questions on this form.**
